# Efficacy of aqueous olanexidine compared with alcohol-based chlorhexidine for surgical skin antisepsis regarding the incidence of surgical-site infections in clean-contaminated surgery: a randomized superiority trial

**DOI:** 10.1093/bjs/znaf065

**Published:** 2025-04-01

**Authors:** Masashi Takeuchi, Hideaki Obara, Tasuku Furube, Hirofumi Kawakubo, Minoru Kitago, Koji Okabayashi, Hiroto Fujisaki, Junya Aoyama, Yosuke Morimoto, Ryusuke Amemiya, Junichi Sano, Jumpei Nakadai, Rei Goto, Yasunori Sato, Yuko Kitagawa

**Affiliations:** Department of Surgery, Keio University School of Medicine, Tokyo, Japan; Department of Surgery, Keio University School of Medicine, Tokyo, Japan; Department of Surgery, Keio University School of Medicine, Tokyo, Japan; Department of Surgery, Keio University School of Medicine, Tokyo, Japan; Department of Surgery, Keio University School of Medicine, Tokyo, Japan; Department of Surgery, Keio University School of Medicine, Tokyo, Japan; Department of Surgery, Hiratsuka City Hospital, Kanagawa, Japan; Department of Surgery, Saiseikai Yokohamashi Tobu Hospital, Kanagawa, Japan; Department of Surgery, Saiseikai Yokohamashi Tobu Hospital, Kanagawa, Japan; Department of Surgery, Kawasaki Municipal Hospital, Kanagawa, Japan; Department of Gastrointestinal Surgery, Saitama City Hospital, Saitama, Japan; Department of Gastrointestinal Surgery, Saitama City Hospital, Saitama, Japan; Graduate School of Business Administration, Keio University, Yokohama, Japan; Department of Biostatistics, Keio University School of Medicine, Tokyo, Japan; Department of Surgery, Keio University School of Medicine, Tokyo, Japan

## Abstract

**Background:**

Surgical-site antisepsis is used to prevent surgical-site infections (SSIs). Although several guidelines have indicated the efficacy of antiseptics, such as chlorhexidine, povidone-iodine, and olanexidine, in reducing the SSI rate, an optimal recommendation is still not established. The aim of this study was to evaluate the efficacy of aqueous olanexidine compared with chlorhexidine-alcohol as the optimal antiseptic for preventing SSI in clean-contaminated surgery.

**Methods:**

This multicentre randomized trial for surgical skin antisepsis in clean-contaminated gastrointestinal and hepatobiliary-pancreatic surgeries in five hospitals evaluated the efficacy of olanexidine and chlorhexidine-alcohol. The primary endpoint was 30-day SSI. Secondary outcomes included the occurrence of SSI types, intervention-related toxicity, and reoperation caused by SSI.

**Results:**

Overall, 700 patients from five institutions underwent randomization; 347 received olanexidine and 345 received chlorhexidine-alcohol in the full analysis set. The 30-day SSI rate was 12.4% (43 of 347) in the olanexidine group and 13.6% (47 of 345) in the chlorhexidine-alcohol group (adjusted risk ratio (aRR) 0.911 (95% c.i. 0.625 to 1.327); *P* = 0.626). No significant differences were observed between the groups regarding the secondary outcomes, including the occurrence of superficial incisional SSI, deep incisional SSI, organ/space SSI, and reoperation caused by SSI. Overall adverse effects were seen in two patients (0.58%) in the olanexidine group and in three patients (0.87%) in the chlorhexidine-alcohol group (aRR 0.663 (95% c.i. 0.111 to 3.951)).

**Conclusion:**

Olanexidine did not significantly reduce the occurrence of overall SSI compared with chlorhexidine-alcohol. Nevertheless, these findings provide valuable insights for developing novel surgical SSI management protocols.

**Registration number:**

UMIN 000049712 (University Hospital Medical Information Network Clinical Trials Registry).

## Teaser

This multicentre, prospective, randomized trial evaluated the efficacy of 1.5% olanexidine and 1.0% chlorhexidine-alcohol for surgical skin antisepsis during clean-contaminated gastrointestinal and hepatobiliary-pancreatic surgeries. In this study, olanexidine did not significantly reduce the occurrence of overall SSI compared with chlorhexidine-alcohol.

## Introduction

Surgical-site infections (SSIs) are among the most prevalent hospital-acquired infections in patients undergoing surgery^1^. Gastrointestinal surgery has notably higher SSI rates than cardiothoracic, gynecological, or neurosurgical procedures^2–4^, leading to extended hospital stays, higher healthcare costs, cosmetic issues, and worse long-term outcomes. Therefore, preventing SSI is critical for both patients and surgeons.

Surgical skin antisepsis is crucial for eradicating microbes from the skin before surgery^5^. Globally, povidone-iodine and chlorhexidine-alcohol are the primary antiseptics in use, with several guidelines favouring the latter^6–11^. The US Centers for Disease Control and Prevention (CDC) guidelines recommend using an alcohol-based agent in the absence of contraindications^1^, whereas global guidelines for SSI prevention by the WHO recommend alcohol-based antiseptic solutions based on chlorhexidine^5^. However, a recent meta-analysis revealed that no significant difference was observed in SSI rates between chlorhexidine-alcohol and aqueous povidone-iodine both in overall and clean-contaminated surgeries^12^. Olanexidine (1.5% Olanedine^®^; Otsuka Pharmaceutical Factory, Inc., Tokushima, Japan), a novel antiseptic, has shown strong activity against various bacteria, including drug-resistant strains^13–15^. The authors previously reported that olanexidine significantly reduces the occurrence of overall SSI and superficial incisional SSI compared with aqueous povidone-iodine, which is the most widely used conventional antiseptic in Japan, after clean-contaminated surgery in a multicentre RCT^16,17^. However, alcohol products recommended by the WHO and the Asia Pacific Society of Infection Control were not applied as a comparison, which was a limitation of that trial^5,18,19^. A subsequent meta-analysis emphasized that further investigation is required to verify the efficacy of olanexidine^20^.

The aim of this multicentre RCT was to compare the efficacy of aqueous olanexidine with chlorhexidine-alcohol as the optimal antiseptic for preventing SSI in clean-contaminated surgery.

## Methods

### Trial design and participants

The OEDO (Oedo is a historical name for Tokyo) trial design has been previously published^21^. This multicentre, randomized, open-label, blinded-endpoint controlled trial evaluated the superiority of 1.5% olanexidine to 1.0% chlorhexidine-alcohol as surgical skin antiseptics for preventing SSI in clean-contaminated gastrointestinal and hepatobiliary-pancreatic surgeries. The surgeries were performed at five general centres that perform >500 gastrointestinal surgeries per year (Keio University Hospital (Tokyo, Japan), Saiseikai Yokohamashi Tobu Hospital (Kanagawa, Japan), Kawasaki Municipal Hospital (Kanagawa, Japan), Hiratsuka City Hospital (Kanagawa, Japan), and Saitama City Hospital (Saitama, Japan)).

The trial was designed and independently conducted by Keio University with approval from the Ethics Committee of the Keio University School of Medicine, in accordance with the principles of the Declaration of Helsinki. The trial protocol (approval no. 20221137; 29 November 2022; version 1.1) was approved by the institutional review boards of each participating institution and was registered in the University Hospital Medical Information Network Clinical Trials Registry (UMIN 000049712), which is one of the network members of the Japan Primary Registries Network that meets the WHO registry criteria.

All participants who met the inclusion criteria received the study briefing from the investigators before providing written informed consent. Inclusion criteria were: undergoing elective surgery under general anaesthesia for gastrointestinal and hepatobiliary-pancreatic diseases (oesophagus, stomach, duodenum, small intestine, colorectal, liver, biliary tract, and pancreas) with a class II surgical wound; age ≥18 years; and provision of written informed consent. The exclusion criteria were: allergy to aqueous olanexidine, chlorhexidine-alcohol, or any other alcohol; inability to undergo follow-up 30 days after surgery; active bacterial infection; antibiotic administration the day before surgery, except for preoperative prophylactic antimicrobials in colorectal surgery; undergoing non-elective surgery or surgery requiring antisepsis of mucosal surfaces or surgical wounds; history of asthma; deemed ineligible to participate in the trial by a physician for any other reason; and inability to provide written informed consent.

### Trial procedures

The commercially available surgical skin antiseptic concentration in Japan, 1.5% olanexidine or 1.0% chlorhexidine-alcohol, was applied immediately before surgery. The patients administered 1.5% olanexidine and the patients administered 1.0% chlorhexidine-alcohol were allocated to the experimental group and the control group respectively. At the surgical site, the antiseptic was administered immediately before surgery from the papilla (up to the neck in the case of thoracic surgery, such as oesophagectomy) at the cranial limit to the upper thigh at the caudal limit. The first incision was made at least 3 min after antiseptic administration to allow the skin to dry. A ready-to-use applicator for olanexidine or a brush or compression using pliers for chlorhexidine-alcohol was used to apply the antiseptic concentrically from the inside to the outside three times. Other procedures for preventing SSI in the protocol were as follows: administering 1 g cefazoline for upper gastrointestinal surgeries, 1 g cefmetazole and 2 g kanamycin monosulfate and 1.5 g metronidazole orally on the day before surgery for lower gastrointestinal surgeries, and 3 g sulbactam/ampicillin for hepatobiliary-pancreatic surgeries intravenously within 60 min before incision; utilizing triclosan-coated absorbable monofilament sutures for wound closure; employing impervious plastic single- or double-ring wound protectors; irrigating the wound with 100–500 ml sterile normal saline; maintaining immunosuppressive agents, such as corticosteroids, methotrexate, or tumour necrosis factor (TNF) inhibitors, with preoperative consultation with the prescribing physician; deciding whether to change or maintain the same gloves and surgical instruments during the operation; and maintaining normal body temperature throughout surgery using warming devices. Preoperative hair removal was not performed routinely.

Typically, in Japan, patients are admitted to the hospital 1–4 days before surgery. Informed consent and background characteristics of patients are collected upon admission. Informed consent for both the surgical procedure and participation in the clinical trial is routinely obtained the day before surgery followed by randomization.

### Randomization and masking

The CapTool^®^ Cloud web-based electronic data capture (EDC) system produced by Mebix Inc. (Tokyo, Japan) was used to register all participants by non-blinded investigators. Patients were randomized to receive skin antisepsis with either 1.5% olanexidine or 1.0% chlorhexidine-alcohol in a 1 : 1 ratio using the EDC system. Block randomization produced by a computer was used to generate random sequences. The allocation was adjusted according to the surgical approach to address the varying SSI rates between laparotomy and laparoscopy, including robotic surgery. The outcome of randomization for each patient was recorded in the electronic case report form. Block sizes were randomly varied, with sizes of four participants, although investigators were not informed of the specific block size during the trial. The allocation manager, Mebix Inc., Tokyo, Japan, devised the allocation procedures and oversaw the allocation data of patients. Allocation data were securely stored and were only accessible to authorized individuals. Patients and investigators who were part of the external evaluation committee for SSI judgement were blinded regarding group allocation. The patients were diagnosed with an SSI or an adverse event based on a completed questionnaire regarding the surgical wound condition completed by non-blinded investigators.

### Trial outcomes

The primary endpoint was 30-day SSI after surgery based on the criteria defined by the CDC guidelines^1^. A superficial incisional SSI was characterized as an infection confined to the skin incision and subcutaneous tissue. A deep incisional SSI was defined as an infection extending into the deeper soft tissues of the incision, involving the fascia or muscle layers. An organ/space SSI was defined as an infection affecting any area of the body accessed or manipulated during the surgical procedure, excluding the incision site and superficial or deep tissue layers. The secondary outcomes were the occurrence of superficial incisional SSI, deep incisional SSI, organ/space SSI, intervention-related toxicity and allergic events (such as erythema, pruritus, dermatitis, or symptoms of allergy around the region disinfected by the antiseptic during surgery), and reoperation caused by SSI. Cost analysis is not reported in the present manuscript. The surgical wound site for each participant was observed daily by non-blinded investigators during admission. After discharge, participants underwent at least one outpatient visit within 30 days of surgery. They were also instructed to visit an outpatient or emergency department whenever symptoms indicative of an SSI, such as pain or redness, were observed.

### Statistical analysis

In a previous meta-analysis, the SSI rate for class II wounds disinfected with chlorhexidine-alcohol was approximately 13.0%^12^, whereas that in the present study was predicted to be approximately 6.5% according to the authors’ previous findings^16^. Using a two-sided chi-squared test at a 5% significance level, a difference in the SSI proportion between olanexidine and chlorhexidine-alcohol was detected with a power of >80%, assuming a group difference of 6.5% over the course of the study. A dropout rate of approximately 10% was permitted. Therefore, the trial required a total sample size of 700 patients (350 in each group).

Primary analyses were performed on the full analysis set (FAS) following the ICH E9 recommendations, from which patients who did not undergo surgery or withdrew consent before the assessment of the primary endpoint were excluded. This approach ensures that the analysis remains as close as possible to the intention-to-treat (ITT) ideal, while accommodating practical constraints. The exclusion criteria for the FAS, such as not having received treatment or a lack of post-randomization data, were strictly predefined and implemented without bias, as recommended by the guidelines. Sensitivity analyses were performed on the per-protocol set, excluding patients with major protocol deviations. ITT analysis was also performed. The safety analysis set included all patients randomly assigned to the study group who received treatment during the study interval. For the baseline variables, summary statistics are presented as *n* (%) for categorical data and as median (range) for continuous data. For the primary analysis, which aimed to compare the treatment effects, the adjusted risk ratio (aRR) and its 95% confidence interval were estimated using the Mantel–Haenszel method. The Mantel–Haenszel test was applied to analyse a significant association with the primary endpoint after adjusting for the allocation factor. All comparisons were planned and all *P* values are two-sided. Statistical significance was set at *P* < 0.050. All statistical analyses were performed using SAS software (SAS Institute, Cary, NC, USA; version 9.4). The principal investigator and biostatistician developed the SAP before patient recruitment and data collection.

## Results

### Patients

**Fig. 1:**
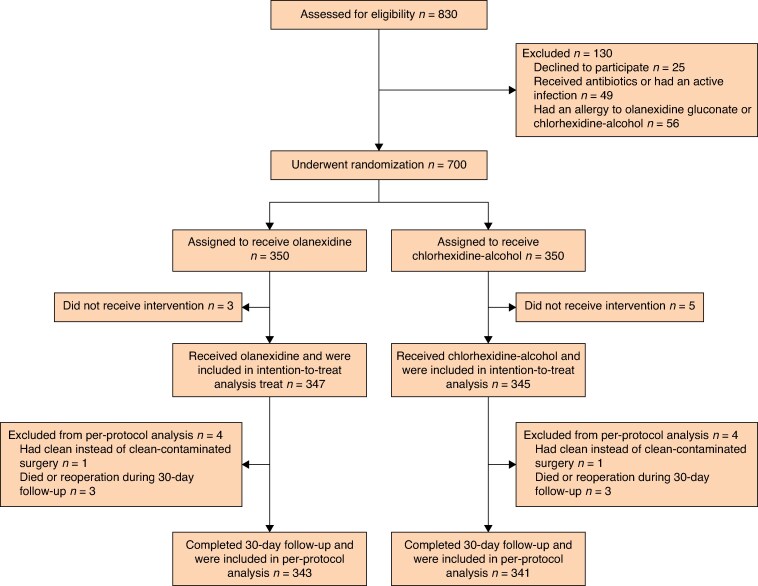
Flow chart of study participants who underwent screening, randomization, and follow-up

Overall, 830 patients were assessed for eligibility between January 2023 and November 2023, with 700 patients from five institutions randomized to the olanexidine group (350 patients) and the chlorhexidine-alcohol group (350 patients) and included in the ITT analysis. Out of the 830 patients, 130 were excluded because they met an exclusion criterion (25 declined to participate, 49 received antibiotics or had an active infection, and 56 had an allergy to olanexidine gluconate or chlorhexidine-alcohol). There were three patients in the olanexidine group and five patients in the chlorhexidine-alcohol group who did not receive the allocated intervention post-randomization, resulting in an FAS of 347 patients in the olanexidine group and 345 patients in the chlorhexidine-alcohol group (*Fig. 1*).

In the olanexidine group, the median age was 69 years and 60.2% were male (209 patients), and, in the chlorhexidine-alcohol group, the median age was 72 years and 58.0% were male (200 patients). Among all patients, 335 underwent hepatobiliary-pancreatic surgery (156 in the olanexidine group and 179 in the chlorhexidine-alcohol group), 200 underwent upper gastrointestinal surgery (111 in the olanexidine group and 89 in the chlorhexidine-alcohol group), and 144 underwent lower gastrointestinal surgery (73 in the olanexidine group and 71 in the chlorhexidine-alcohol group). Laparoscopy, including robotic surgery, was performed on 308 patients (88.8%) in the olanexidine group and on 308 patients (89.3%) in the chlorhexidine-alcohol group. There were no statistically significant differences in the baseline demographics (*Table 1* and *Table S1*).

**Table 1:** Patient demographics for the olanexidine group and the chlorhexidine-alcohol group

	Olanexidine (*n* = 347)	Chlorhexidine-alcohol (*n* = 345)
**Sex**		
Male	209 (60.23)	200 (57.97)
Female	138 (39.77)	145 (42.03)
**Age (years), median (range)**	69 (24–91)	72 (19–92)
**Current smoking**	86 (24.78)	90 (26.16)
**ASA grade**		
I	124 (35.73)	130 (37.68)
II	194 (55.91)	180 (52.17)
III	29 (8.36)	35 (10.14)
**Diabetes mellitus**	61 (17.58)	68 (19.71)
**Albumin <3 g/dl**	17 (4.90)	29 (8.41)
**Steroid used**	3 (0.86)	6 (1.74)
**Surgery**		
Upper gastrointestinal	111 (31.99)	89 (25.80)
Lower gastrointestinal	73 (21.04)	71 (20.58)
Hepatobiliary-pancreatic	156 (44.96)	179 (51.88)
Other	7 (2.02)	6 (1.74)
**Laparoscopy**	308 (88.76)	308 (89.28)
**Intraoperative blood loss (ml), median (range)**	25 (0–1082)	25 (0–2217)

Values are *n* (%) unless otherwise indicated.

### Primary endpoint and secondary outcomes

Regarding the primary endpoint of the FAS, 43 patients (12.3%) and 47 patients (13.6%) were diagnosed with SSI in the olanexidine group and the chlorhexidine-alcohol group respectively (aRR 0.911 (95% c.i. 0.625 to 1.327); adjusted risk difference −0.012 (95% c.i. −0.061 to 0.037); *P* = 0.626) (*Table 2*). No significant differences were observed between the olanexidine group and the chlorhexidine-alcohol group regarding the secondary outcomes, including the occurrence of superficial incisional SSI (17 patients (4.9%) *versus* 19 patients (5.5%) respectively; aRR 0.890 (95% c.i. 0.472 to 1.681)), deep incisional SSI (3 patients (0.9%) *versus* 5 patients (1.5%) respectively; aRR 0.597 (95% c.i. 0.144 to 2.475)), organ/space SSI (23 patients (6.6%) *versus* 23 patients (6.7%) respectively; aRR 0.996 (95% c.i. 0.576 to 1.721)), and reoperation caused by SSI (2 patients (0.6%) *versus* 4 patients (1.2%) respectively; aRR 0.500 (95% c.i. 0.092 to 2.710)). There were no significant differences in overall adverse effects between the olanexidine group and the chlorhexidine-alcohol group (two patients (0.6%) *versus* three patients (0.9%) respectively; aRR 0.663 (95% c.i. 0.111 to 3.951)). The per-protocol analysis showed the same tendency as that of the FAS between the two groups (*Table S2*). All analyses were performed using the ITT population with similar results to the FAS (*Table S3*).

**Table 2:** Pre-specified primary endpoint and secondary outcomes according to the intervention group in the FSA

	Olanexidine (*n* = 347)	Chlorhexidine-alcohol (*n* = 345)	Adjusted risk difference (95% c.i.)	Adjusted risk ratio (95% c.i.)	*P*
**Primary endpoint**					
SSI	43 (12.39)	47 (13.62)	−0.012 (−0.061,0.037)	0.911 (0.625,1.327)	0.626
**Secondary outcomes**					
Type of SSI					
Superficial incisional	17 (4.9)	19 (5.51)	−0.006 (−0.039,0.027)	0.890 (0.472,1.681)	
Deep incisional	3 (0.86)	5 (1.45)	−0.006 (−0.022,0.010)	0.597 (0.144,2.475)	
Organ/space	23 (6.63)	23 (6.67)	−0.0003 (−0.037,0.036)	0.996 (0.576,1.721)	
Adverse skin reaction					
All	2 (0.58)	3 (0.87)	−0.003 (−0.016,0.010)	0.663 (0.111,3.951)	
Erythema	0 (0)	3 (0.87)	−0.009 (−0.019,0.001)	0.142 (0.007,2.736)*	
Pruritus	1(0.29)	0 (0)	0.003 (−0.003,0.009)	3.000 (0.126,71.40)*	
Dermatitis	2 (0.58)	0 (0)	0.006 (−0.002,0.014)	2.990 (0.315,28.39)*	
Reoperation caused by SSI	2 (0.58)	4 (1.16)	−0.006 (−0.020,0.0080)	0.500 (0.092,2.710)	

Values are *n* (%) unless otherwise indicated. *Use of continuity correction of 0.5 for zero-event outcome. FAS, full analysis set; SSI, surgical-site infection.

Overall, 90 patients in the present study experienced SSI. Out of these 90 patients, 10 had more than one type of SSI. Four patients with an infection with subcutaneous tissue and organ/space involvement, one patient with an infection with superficial, subcutaneous tissue, and organ/space involvement, and four patients with an infection with superficial and organ/space involvement were classified as having organ/space SSI. One patient with an infection with superficial and subcutaneous tissue involvement was classified as having deep incisional SSI.

### Subgroup analysis

**Fig. 2:**
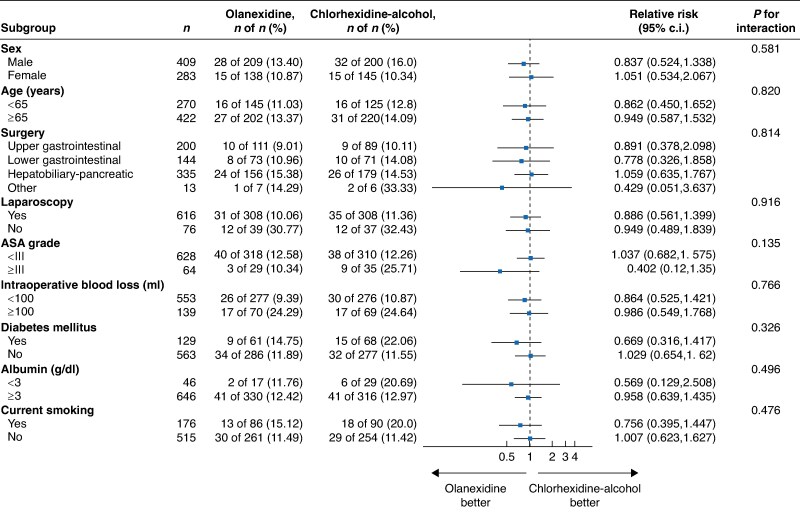
Subgroup analysis of overall SSI in the FAS Interaction *P* values indicate whether the effect of antiseptics on SSI differed for each subgroup. SSI, surgical-site infection; FAS, full analysis set.

Whether the SSI rate was correlated with the type of skin antiseptic used was examined across various subgroups. These subgroups were based on the following: sex, age, type of surgery, use of laparoscopy, ASA grade, intraoperative blood loss, presence of diabetes mellitus, albumin level, and smoking habits. There were no significant interactions between these factors and SSI rates, indicating that the efficacy of the two antiseptics did not vary significantly across the subgroups (*Fig. 2*).

### Bacterial strains

A positive wound bacterial test was observed in 49 patients in the olanexidine group and 41 patients in the chlorhexidine-alcohol group (*Table S4*). The pathogen was identified in 35 patients (71.4%) in the olanexidine group and 34 patients (82.9%) in the chlorhexidine-alcohol group. The most common organisms cultured from surgical sites were *Enterococcus* spp. (12 of 49 (24.5%) in olanexidine group and 12 of 41 (29.3%) in chlorhexidine-alcohol group). For some patients, more than one organism was cultured; moreover, for some patients there were no cultures or negative cultures.

## Discussion

This multicentre randomized superiority trial found no significant difference in the occurrence of SSI between the olanexidine group and the chlorhexidine-alcohol group, indicating that the efficacy of olanexidine in preventing overall SSI in clean-contaminated surgery is comparable to that of chlorhexidine-alcohol. To the authors’ knowledge, this study is the first to compare these antiseptics in a multicentre RCT setting and adds critical evidence to the ongoing debate on the optimal antiseptic choice for clean-contaminated surgeries.

Several prospective studies have assessed the efficacy of olanexidine. In addition to the authors’ previous study^16^, Umemura *et al*.^22^ reported an overall SSI rate of 12.8% and an incisional SSI rate of 6.7% when using olanexidine in gastroenterological surgery and Iida *et al*.^23^ also showed similar results for olanexidine, with overall and superficial incisional SSI rates of 10.2% and 2.0% respectively. In the present trial, the SSI rate was approximately 13% and the superficial incisional SSI rate was approximately 5% both in the olanexidine group and the chlorhexidine-alcohol group, in line with the findings of previous studies from other institutions. However, these rates were higher than those in the authors’ previous trial^16^. This may be attributed to significant differences in patient backgrounds, even though both studies focused on clean-contaminated surgery. For instance, the proportion of patients with an ASA grade of ≥III was approximately 4% in the previous study^16^, but increased to approximately 10% in the present study. Additionally, the number of malnourished patients more than doubled, to approximately 7%, and the percentage of smokers also doubled, to >25%. The participating facilities also differed and all of these variations in background factors may have influenced the results^24–26^.

Several prospective studies and meta-analyses have found chlorhexidine-alcohol to be an effective antiseptic for preventing SSI, yet the most effective antiseptic has not been established and SSI remains a common complication after clean-contaminated surgeries^20,27^. The authors’ previous RCT^16^ assessed olanexidine *versus* povidone-iodine, Japan’s preferred antiseptic, focusing on SSI prevalence. The next step was to compare chlorhexidine-alcohol and olanexidine, which are superior to povidone-iodine. The main advantage of the present trial was that it used a blinded approach to diagnose SSI at multiple centres and incorporated only major centers that performed >500 gastrointestinal and hepatobiliary-pancreatic surgeries annually, ensuring consistent quality of care. Furthermore, standardized SSI management was coordinated by the SSI management team at Keio University to ensure uniformity across facilities.

Nishioka *et al*.^15^ investigated the fast-acting bactericidal activity and substantivity of olanexidine compared with other antiseptics, such as chlorhexidine-alcohol, using an *ex vivo* Yucatan micropig skin model and found that olanexidine showed similar activity to or stronger activity than chlorhexidine-alcohol and povidone-iodine. Thus, the efficacy of olanexidine was expected and a significant difference might have been found if more patients had been included. An advantage of olanexidine is that it can be applied using a ready-to-use applicator, the design of which is particularly effective for relatively flat areas, such as those encountered in abdominal surgeries. Typically, antisepsis requires beakers, brushes, or sponges, such as when using chlorhexidine-alcohol, which can ultimately lead to higher costs. Additionally, issues such as flammability and allergies associated with alcohol can be resolved using olanexidine.

In the present study, a lower concentration of chlorhexidine (1.0%) was used as a control, as it is the concentration that is available in Japan. Due to the risk of anaphylaxis, Japanese pharmaceutical regulations limit the concentration of chlorhexidine in skin antisepsis to a maximum of 1.0%. Although no studies have directly compared the various concentrations of chlorhexidine, meta-analyses have indicated that the risk ratio of SSI for 0.5% and 2.0% chlorhexidine-alcohol is significantly lower than that for povidone-iodine, suggesting the effectiveness of low concentrations of chlorhexidine^28^. However, a new study may be required to verify the superiority of olanexidine to 2.0% chlorhexidine-alcohol in other countries in which a concentration >1.0% chlorhexidine may be applied.

There are limitations regarding the present trial. First, variations in patient demographics and surgical practices across sites, along with the exclusive use of Japanese patients due to the limited global availability of olanexidine, may limit the generalizability of the findings. Second, the inclusion of a wide array of gastroenterological procedures introduced variability in the inherent SSI risks and patient stratification was not performed at the hospital site. However, as all surgeons at Keio University are educated about SSI management and the facilities were capable of managing SSI similarly, the effect of this bias is minimal.

In conclusion, olanexidine was not superior to chlorhexidine-alcohol in reducing SSI rates, but it had comparable efficacy, supporting its use as an alternative antiseptic in clean-contaminated surgeries, particularly in the context of contraindicated alcohol-based solutions.

## Supplementary Material

znaf065_Supplementary_Data

## Data Availability

Individual participant data that underlie the results reported in this article will be available after de-identification. The study protocol and statistical analysis plan will also be available to investigators whose proposed use of the data has been approved by an independent review committee identified for this purpose. Proposals should be directed toward obara.z3@keio.jp. Data requestors must sign data-access agreements to gain access. Data will be available for 5 years on a third-party website.
