# Supplementary material for: Efficacy of aqueous olanexidine compared with alcohol-based chlorhexidine for surgical skin antisepsis regarding the incidence of surgical-site infections in clean-contaminated surgery: a randomized superiority trial
Source: Br J Surg. 2025 Apr 1;112(4):znaf065. doi: 10.1093/bjs/znaf065 (PMC12475902; doi:10.1093/bjs/znaf065)
Supplement: znaf065_Supplementary_Data [file znaf065_supplementary_data.zip › Statistical_analysis_plan.docx]

**The efficacy of aqueous olanexidine compared to alcohol-based chlorhexidine for surgical skin antisepsis on incidence of surgical site infections in clean-contaminated surgery: a randomised superiority trialTracking Number: UMIN 000049712**

**STATISTICAL ANALYSIS PLAN**

**Ver 2.0**

#### PRINCIPAL INVESTIGATOR：

Department of Surgery, Keio University, School of Medicine

Hideki Obara, MD.PhD.

Approved on 10/January/2023

Revised on 06/January/2025

#### COORDINATING INVESTIGATOR：

Department of Surgery, Keio University, School of Medicine

Masashi Takeuchi, MD.PhD.

**TRIAL STATISTICIAN**

Department of Preventive Medicine and Public Health, Keio University School of Medicine

Yasunori Sato, PhD

1. GENERAL CONVENTIONS IN STATISTICAL ANALYSIS
   1. Interim Analysis

No interim analysis will be done in this trial.

- 1. Data Monitoring Committee

Data Monitoring Committee will be held to ensure the safety of the trial subjects in accordance with a separate set of operating procedure.

- 1. Handling of Missing Data

Unless otherwise noted, withdrawn or missing data will not be replaced with substituted values created by statistical imputation.

- 1. Data Transformation

In case of change of variables such as square root or logarithm, precise procedure will be described in the statistical analysis plan.

- 1. Significance Level and Confidence Interval

Unless otherwise noted, results from all analysis will be assessed against the significance level(α) of 0.05 and the confidence interval (CI) of 0.95 as two-sided test.

- 1. Multiplicity Adjustment

Unless otherwise noted, statistical adjustments for multiplicity will not be performed in this trial.

- 1. Subgroup Analysis

Not applicable

- 1. Data Processing Conventions

**Definitions of days and period**

- In calculation of the number of days, the beginning date will be subtracted from the final date and then one day will be added.

(examples)

- Days dosed: If dosing is ended on the first date dosed , the dosing period will be counted as one day.
- Days to the occurrence of an adverse event(AE): If an AE occurs next day after the first date dosed, the period to the onset of the AE will be counted as two days.
- Survival period：If death occurs next day after the first date dosed, the survival period will be counted as two days.
- The number of days will be calculated as follows:
- One year: 365.25 days
- One month:30.4375 days
- One week: 7 days
- In counting the number of days after the initial date as follows:
- The initial date dosed: day (1)
- Next day after the initial date: day(2)
- Two days after the initial date: day(3)

The following days will be treated as the same.

- One day before the initial date dosed: day (-1)
- Two days before the initial date: day(-2)
- Three days before the initial date: day(-3)

The following days will be treated as the same.

**Handling of digits after the decimal point**

- One digit lower than the raw data values will be added to provide the values of mean, standard deviation, and median. (The values will be rounded at the next digit.)
- All percentages should be rounded and reported at a single decimal place (e.g.12.3%)。
- In calculating mean, standard deviation, and/or median for statistical analysis, rounding will not be performed before the final calculation but only for the final results.

**Confidence Interval (CI) for the incidence rate**

Confidence interval of incidence rate (or incidence percentage), response rate (or response percentage) will be calculated by correct method with F distribution.

The number of population for analysis will be defined as N, and the number of patients in whom the events occurred, or the number of responders to the treatment as X. The response rate*(p)* , the upper limits (*pu*), or lower limits(*p_L_*) of the confidence interval of 100 (1 – *α*)% at a two sided-test, will be calculated by the following equations. 95% confidence interval reflects α of 0.05.

$$p=\frac{X}{N},$$

$$p_{U}=\left( 1+\frac{N-X}{(X+1)F\left( 1-\frac{\alpha}{2},2(X+1),2\left( N-X \right) \right)} \right)^{-1},$$

$$p_{L}=\left( 1+\frac{N-X+1}{X F\left( 1-\frac{\alpha}{2},2X,2\left( N-X+1 \right) \right)} \right)^{-1}.$$

1. ANALYSIS
   1. Definitions of Population
      1. Full Analysis Set (FAS)

FAS is defined as all enrolled subjects who do not undergo surgery or who withdrew consent before the assessment of the primary endpoint will be excluded.

- - 1. Per Protocol Set (PPS)

The PPS will exclude all subjects in the FAS population who meet any of the following major violations of the protocol.

- Inclusion criteria violation
- Exclusion criteria violation
- Prohibited concomitant drug use violation
- Prohibited concomitant therapy violation
  - 1. Intention-to-trea Set (ITT)

The ITT will include all subjects who were randomly assigned.

- - 1. Safety Analysis Set (SAF)

Safety analyses will be performed on the SAF population subjects who are enrolled in the trial and applied the assigned disinfectant at least once for each treatment group.

- 1. Analysis Principles

The primary analysis for all efficacy endpoints will be performed on the FAS while the analyses on the PPS and ITT will be treated as reference data. Safety analyses will be performed on the SAF.

1. **DEMOGRAPHIC AND BASELINE CHARACTERISTICS**

Demographic and other baseline characteristic will be summarized by assigned treatment group with descriptive statistics. Continuous variables will be summarized with demographic statistics (number of patients(n), mean, standard deviation, min, max, and median).

Categorical variables will be summarized by frequencies and percentages according to assigned treatment group. A t-test or Wilcoxon’s rank-sum test will be used for comparison of treatment group with continuous variables. Fisher’s exact test will be used with categorical data.
Results from all analysis will be assessed against the significance level (α) of 0.05 at two-sided test.

1. ANALYSIS OF EFFICACY
   1. Analysis Plan for Primary Endpoints

Primary efficacy endpoint is postoperative 30-day SSI. To compare the treatment effects, the adjusted risk ratio and its 95% CI will be estimated using the Mantel-Haenszel method. To test for a significant association with the primary outcome, the Mantel-Haenszel test will be applied after the adjustment for allocation factors.

- 1. Analyses Plan for Secondary Endpoints

The analyses of the secondary efficacy endpoints will be performed to support the discussion on the results of the primary analysis in this trial. Multiplicity adjustment will not be performed in this trial in assessing secondary endpoints.

To compare the treatment effects, the adjusted risk ratio and its 95% CI will be estimated using the Mantel-Haenszel method. To test for a significant association with the primary outcome, the Mantel-Haenszel test will be applied after the adjustment for allocation factors.

Secondary efficacy endpoints

The Mantel-Haenszel test will be applied after adjusting for allocation factors.

1. **Postoperative 30-day superficial incisional SSI rate**
2. **Deep incisional SSI rate, organ/space SSI rate**
3. **Rate of positive bacterial wound culture, and their strains**
4. **Bacterial strain**
5. **Toxicity and allergies events (erythema, symptoms of allergy) rate.**
6. **Reoperation rate due to SSI**
7. SUBGROUP ANALYSIS
   1. Plan for Subgroup Analysis

Subgroup analysis for “Analysis of Efficacy” or “Analysis of Safety” will be performed. We conducted ten (male versus female, age≧65 year-old versus age< 65 year-old, upper gastrointestinal surgery versus lower gastrointestinal surgery versus hepatobiliary surgery, laparoscopy versus laparotomy, obese (Body mass index≧25 kg/m^2^) versus non-obese, ASA≧3 versus ASA<3, blood loss≧100ml versus blood loss<100ml, diabetic versus non-diabetics, malnutrition versus non- malnutrition, smoking versus non-smoking ) subgroup analyses for the primary outcome and secondary outcome (postoperative 30-day superficial incisional SSI) using the Mantel-Haenszel test.

1. REVISION HISTORY
   1. Changes According to Protocol Amendment

| Date | Name | Distribution | Changes |
| --- | --- | --- | --- |
| - | - | - | - |

- 1. Revision History of Statistical Analysis Plan

| Date | Name | Distribution | Changes |
| --- | --- | --- | --- |
| 01/10, 2023 | Yasunori Sato | None | Version 1 |
| 01/06,2025 | Yasunori Sato | Added Intention-to-treat set (ITT) to 2.2 Analysis Principles for peer review. The definition of ITT was added to 2.1.3. | Version2 |
|  |  |  |  |

1. STATISTICAL ANALYSIS IMPLEMENTATION SYSTEM
   1. Biostatistical Supervisor

Department of Preventive Medicine and Public Health, Keio University School of Medicine

Yasunori Sato,PhD

- 1. Hardware System

The following hardware version will be used for statistical analysis.

- Microsoft Windows XP or following OS
  1. Software System

The following software will be used for statistical analysis.

- SAS 9.4 or higher
- Microsoft Office 2013or after
- Adobe Acrobat XI or higher
