# Supplementary material for: Efficacy of aqueous olanexidine compared with alcohol-based chlorhexidine for surgical skin antisepsis regarding the incidence of surgical-site infections in clean-contaminated surgery: a randomized superiority trial
Source: Br J Surg. 2025 Apr 1;112(4):znaf065. doi: 10.1093/bjs/znaf065 (PMC12475902; doi:10.1093/bjs/znaf065)
Supplement: znaf065_Supplementary_Data [file znaf065_supplementary_data.zip › Study_protocol.docx]

**The efficacy of aqueous olanexidine compared to alcohol-based chlorhexidine for surgical skin antisepsis on incidence of surgical site infections in clean-contaminated surgery: a multicenter, prospective, randomised, blinded-endpoint superiority trial (OEDO trial)**

**Trial protocol**

Principal Investigator

Hideaki Obara, Department of Surgery (General/Gastrointestinal), Keio University School of Medicine

Managing Investigator

Masashi Takeuchi, Department of Surgery (General/Gastrointestinal), Keio University School of Medicine

　　　35 Shinanomachi, Shinjuku-ku, Tokyo, 160-8582, Japan

　　　TEL：03-5363-3802

　　　FAX：03-3355-4707

E-mail：[masaty871222@gmail.com](mailto:masaty871222@gmail.com)

Mobile: 070-4833-3990

7/31/2022 First edition (ver. 1.0) created

11/29/2022 Ver. 1.1 created

1/5/2023 Ver. 2.0 created

UMIN 000049712

0　Summary

0.1　Outline

Elective gastrointestinal surgery under general anesthesia with class II wounds

Patients aged ≥ 18 years with no allergy to aqueous olanexidine or alcohol-based antiseptics such as chlorhexidine for surgical skin antisepsis

Random assignment

Group A: aqueous olanexidine group

Group B: alcohol-based chlorhexidine group

0.2　Objectives

To compare the surgical site infection (SSI) reduction effect of 1.5% aqueous olanexidine with that of 1% alcohol-based chlorhexidine on surgery involving class II wounds in the gastrointestinal region.

(1) Primary endpoint

Incidence of SSI within 30 days post-operation

(2) Secondary endpoints

Incidence of superficial incisional SSI up to 30 days post-operation, incidence of deep incisional SSI, incidence of organ/space SSI, positive wound culture rate and strains detected in patients who underwent wound culture, incidence of adverse drug reactions (ADRs) (erythema, allergy symptoms, etc.), rate of reoperation due to SSI, medical economic effect indicators (data based on health insurance claims), duration of hospitalization

(3) Exploratory endpoints

Not established in the present trial

(4) Safety endpoints

Incidence of ADRs (erythema, allergy symptoms, etc.)

0.3 Participants

Eligibility criteria

Patients are considered eligible to participate if they meet all of the following conditions.

1. Patients undergoing elective surgery under general anesthesia in the gastrointestinal region (esophagus, stomach, duodenum, small intestine, large intestine, liver, biliary tract, pancreas) with class II wounds
2. Age ≥ 18 years at the time of consent to participate in the trial
3. Provision of written consent from the patient to participate in the trial

Exclusion criteria

Patients who meet any of the following criteria are excluded.

1)　 Patients with a history of allergy to aqueous olanexidine, alcohol-based chlorhexidine, or any other alcohol

2)　Patients unable to undergo 30-day postoperative follow-up

3)　Patients with active infections (except for viral hepatitis)

4)　Patients who use antimicrobials the day before surgery (except for preoperative prophylactic antimicrobials in colorectal surgery)

5)　Emergency surgery or surgery requiring antisepsis of mucosal surfaces or wounds

6) Patients with a history of asthma

7) Patients deemed by a physician to be ineligible to participate in the trial safely for any other reason

0.4　Investigational treatments

Group A: Use of 1.5% aqueous olanexidine to disinfect the operative field immediately before surgery

Group B: Use of 1% alcohol-based chlorhexidine to disinfect the operative field immediately before surgery

Guidelines for perioperative management are established as follows in accordance with Global Guidelines for the Prevention of Surgical Site Infection.

1. Administer antimicrobials prior to incision
2. Use absorbable sutures to close wounds. Antibacterial sutures are preferable
3. The use of a wound retractor in laparotomy is irrelevant but preferable
4. Saline irrigation of wounds prior to closure is preferable but not required
5. The use versus non-use of immunosuppressants is irrelevant
6. Changing versus no change of gloves prior to wound closure is irrelevant
7. Changing versus no change of surgical implements prior to wound closure is irrelevant

0.5　Target sample size and scheduled study period

Number of patients enrolled: 700

Study period: Date of IRB approval – 12/31/2025

0.6　Inquiries

Study Secretariat

Department of Surgery (General/Gastrointestinal), Keio University School of Medicine

35 Shinanomachi, Shinjuku-ku, Tokyo, 160-8582, Japan5

TEL：03-5363-3802　FAX：03-3355-4707

E-mail：[masaty871222@gmail.com](mailto:masaty871222@gmail.com)

PHS：070-4833-3990

Masashi Takeuchi

Table of contents

[1　Summary 5](#_Toc460432923)

[2　Background information 7](#_Toc460432931)

[3　Objectives 9](#_Toc460432937)

[4　Trial design 10](#_Toc460432944)

[5　Participant inclusion/exclusion/withdrawal criteria 15](#_Toc460432947)

[6　Investigational treatments 16](#_Toc460432949)

[7　Efficacy assessment 17](#_Toc460432953)

[8　Safety assessment 18](#_Toc460432958)

[9　Statistical analysis 20](#_Toc460432960)

[10　Source data verification for raw data and source documents 21](#_Toc460432963)

[11　Monitoring and auditing 22](#_Toc460432966)

[12　Ethical considerations 22](#_Toc460432967)

[13　Handling of data/samples and preservation of records 24](#_Toc460432970)

[14　Economic burden on participants and measures other than insurance 24](#_Toc460432973)

[15　Arrangements regarding publication of trial results 24](#_Toc460432974)

[16　Supplement 24](#_Toc460432991)

[17　References 24](#_Toc460432992)

**1　Summary**

1.1　Trial information

The objective of the present trial is to compare the SSI reduction effect of 1.5% aqueous olanexidine with that of 1% alcohol-based chlorhexidine on class II wounds in surgery in the gastrointestinal region.

Fig: CDC wound classifications

・Class I (clean): No inflammation; uninfected wounds that do not enter respiratory, alimentary, genital, or urinary tracts.

・Class II (clean-contaminated): No inflammation; wounds that have entered the respiratory, alimentary, genital, or urinary tracts, are well-controlled, and lack unusual contamination.

・Class III (contaminated): Fresh, open, accidental wounds.

・Class IV (dirty-infected): Old trauma with persistent necrotic tissue and old trauma with clinical infection or perforated viscera.

1.2　Principal investigator

Hideaki Obara, Department of Surgery (General/Gastrointestinal), Keio University School of Medicine

35 Shinanomachi, Shinjuku-ku, Tokyo, 160-8582, Japan

TEL：03-5363-3802

FAX：03-3355-4707

E-mail：[obara.z3@keio.jp](mailto:obara.z3@keio.jp)

l.3　Study secretariat

Masashi Takeuchi, Department of Surgery (General/Gastrointestinal), Keio University School of Medicine

35 Shinanomachi, Shinjuku-ku, Tokyo, 160-8582, Japan

TEL：03-5363-3802　FAX：03-3355-4707

E-mail：[masaty871222@gmail.com](mailto:masaty871222@gmail.com)

PHS：070-4833-3990

1.4 Medical expert

In the present study, the role of medical expert who makes medical judgments during the trial was performed by the principal investigator.

1.5 Outsourcing facilities

The construction of an electronic data capture system and the management of that data are outsourced to Mebix, Inc.

1.6 Trial sites and investigators

Trial site Investigator (title)

Department of Surgery (General/Gastrointestinal), Keio University School of Medicine Hideaki Obara (Associate Professor)

35 Shinanomachi, Shinjuku-ku, Tokyo, 160-8582, Japan

TEL：03-5363-3802　FAX：03-3355-4707

Department of General and Gastrointestinal Surgery, Kawasaki Municipal Hospital　Masaya Shito (Department Director)

12-1 Shinkawadori, Kawasaki-ku, Kawasaki, Kanagawa, 210-0013, Japan

TEL：044-233-5521 FAX：044-245-9600

Department of Gastrointestinal Surgery, Saiseikai Yokohamashi Tobu Hospital Tomohisa Egawa (Assistant Director)

3-6-1 Shimosueyoshi, Tsurumi-ku, Yokohama, Kanagawa, 230-8765, Japan

TEL: 045-576-3000 FAX: 045-576-3547

Department of Surgery, Hiratsuka City Hospital

Motohito Nakagawa (Assistant Hospital Director)

1-19-1 Minamihara, Hiratsuka, Kanagawa, 254-0065, Japan

TEL: 0463-32-0015 FAX: 0463-32-0015

Department of Gastrointestinal Surgery, Saitama City Hospital

Hideo Baba (Department Director)

2460 Mimuro, Midori-ku, Saitama, Saitama, 336-8522, Japan

TEL：048-873-4111 FAX: 048-873-5451

1.7　Monitor

Kazumasa Fukuda, Lecturer, Department of Surgery (General/Gastrointestinal), Keio University School of Medicine

35 Shinanomachi, Shinjuku-ku, Tokyo, 160-8582, Japan

TEL：03-5363-3802

FAX：03-3355-4707

E-mail：[fukudak@z6.keio.jp](mailto:fukudak@z6.keio.jp)

1.8　Analysts

Yasunori Sato, Associate Professor, Clinical & Translational Research Center, Keio University School of Medicine

35 Shinanomachi, Shinjuku-ku, Tokyo, 160-8582, Japan

TEL：03-5363-3802

FAX：03-3355-4707

E-mail：[yasunori.sato@keio.jp](mailto:yasunori.sato@keio.jp)

Rei Goto, Professor, Keio University Graduate School of Health Management

Kyoseikan, 4-1-1 Hiyoshi, Kohoku-ku, Yokohama, 223-8526, Japan

TEL：045-564-2536

FAX：045-564-2536

E-mail：[reigoto@kbs.keio.ac.jp](mailto:reigoto@kbs.keio.ac.jp)

Takashi Yoshioka, Project Assistant, Department of Preventive Medicine and Public Health, School of Medicine, Keio University

35 Shinanomachi, Shinjuku-ku, Tokyo, 160-8582, Japan

TEL: 03-5363-3758

FAX: 03-3359-3686

E-mail: ty5733@keio.jp

Satoshi Funada (Project Researcher), Department of Preventive Medicine and Public Health, School of Medicine, Keio University

35 Shinanomachi, Shinjuku-ku, Tokyo, 160-8582, Japan

TEL: 03-5363-3758

FAX: 03-3359-3686

E-mail:sfunada@kuhp.kyoto-u.ac.jp

1.9 External Evaluation Committee for SSI Judgement

Yusuke Maeda, Department of Surgery, Akasakamitsuke Maeda Hospital

1-1-5 Moto-Akasaka, Minato-ku, Tokyo 107-0051, Japan

TEL: 03-3408-1136 FAX: 03-3408-8566

Yasuhito Sekimoto, Department of Surgery, National Hospital Organization Tokyo Medical Center

2-5-1 Higashigaoka, Meguro-ku, Tokyo 152-8902, Japan

TEL: 03-3411-0111 FAX: 03-3412-9811

Takayuki Kondo, Tokatsu Tsujinaka Hospital

946-1 Nedo, Abiko, Chiba 270-1168, Japan

TEL：047-184-9000

**2　Background**

2.1　Investigational agents (investigational equipment, investigational treatments)

Olanedine® solution 1.5% OR antiseptic applicator 25 mL (Otsuka Pharmaceutical Factory, Inc.)

Characteristics/Mechanism of action:

Olanedine® solution is a novel biguanide-based antiseptic agent with olanexidine gluconate as its active ingredient. Its mechanism of action is as follows: it demonstrates bactericidal activity by binding to bacterial membranes, damaging the membrane structure and destroying membrane barrier capacity, thereby causing irreversible leakage of cytoplasmic components; in relatively high concentrations, it is believed to agglutinate and kill bacteria via protein denaturation action^1^. A pharmacological experiment found that Olanedine® solution demonstrates antibacterial activity against a broad spectrum of gram-positive and gram-negative bacteria, with particularly potent and fast-acting bactericidal activity against gram-negative bacteria such as MRSA and VRE^2^.

Indication:

Surgical site (operative field) skin antisepsis

Pharmacokinetics:

In patients scheduled to undergo laparoscopic gastrointestinal surgery, levels of serum olanexidine at the time of preoperative application of Olanedine® solution were below the lower limit of quantitation (0.05 ng/mL) in 27 of 52 patients. In the 25 patients for whom levels exceeded the lower limit of quantitation, peak plasma concentration was 0.053−1.536 ng/mL. Olanedine® solution was observed in serum 0.5 hours after application, with mean serum concentration peaking 2 hours after application. At 168 hours after application, levels were below the lower limit of quantitation in all study participants. In healthy individuals, serum levels of olanexidine at the time of abdominal or inguinal application of aqueous olanexidine (1%, 1.5%, 2%) were below the lower limit of quantitation in 64 of 66 participants. In the two participants with levels exceeding the lower limit of quantitation, peak plasma concentration was 0.136 and 0.276 ng/mL.

Major ADRs:

In a phase III clinical trial conducted at 16 Japanese centers with patients scheduled to undergo laparoscopic gastrointestinal surgery, three ADRs were observed in 3 of 52 patients (5.8%) in the safety analysis set. These ADRs consisted of one incidence each of application site dermatitis, application site erythema, and application site pruritus in 1 participant each (1.9%).

2.2　Non-clinical trials and past clinical trials

Two phase III clinical trials were conducted by Otsuka Pharmaceutical Factory as confirmatory trials. In one trial, which was conducted with healthy individuals, the investigational agent was applied once to the abdomen and groin; with bacterial count 10 minutes after application as the primary efficacy endpoint variable, Olanedine® solution was shown to be superior to a placebo control and non-inferior to an active control (0.5% chlorhexidine gluconate solution: CHG), thus confirming Olanedine® solution to be effective and safe. As for ADRs, application site erythema occurred in 1.3% of participants in the Olanedine® solution group (3/237), 0.8% of participants in the CHG group (2/237), and 0.8% of participants in the placebo group (1/120).

In another phase III clinical trial, a multicenter randomized clinical trial which was conducted with patients scheduled to undergo laparoscopic gastrointestinal surgery, either Olanedine® solution or 10% povidone-iodine solution was applied to the skin of the surgical site (operative field); medical comparisons of skin findings in the investigational agent application site, subjective and objective symptoms, vital signs, and laboratory tests confirmed the safety and pharmacokinetics of Olanedine® solution. Levels of serum olanexidine at the time of preoperative skin application of Olanedine® solution were below the lower limit of quantitation (0.05 ng/mL) in 27 of 52 patients. In the 25 patients for whom levels exceeded the lower limit of quantitation, peak plasma concentration was 0.053−1.536 ng/mL. Olanedine® solution was observed in serum 0.5 hours after application, with mean serum concentration peaking 2 hours after application. At 168 hours after application, levels were below the lower limit of quantitation in all study participants. Three ADRs were observed in 3 of 52 patients (5.8%) in the safety analysis set. These ADRs consisted of one incidence each of application site dermatitis, application site erythema, and application site pruritus in 1 participant each (1.9%). The above two trials can be said to have established the safety and efficacy of Olanedine® solution.

In addition, a randomized controlled trial was conducted as a four-center superiority trial by Keio University and three other associated centers. In this trial, patients aged ≥ 20 years undergoing surgery under general anesthesia in the gastrointestinal region (esophagus, stomach, duodenum, small intestine, large intestine, liver, biliary tract, pancreas) with class II wounds were randomly divided into two groups: one which underwent surgical site antisepsis with olanexidine immediately prior to surgery; and one which underwent surgery with 10% povidone-iodine, an iodine antiseptic. A total of 597 patients were enrolled between June 2018 and April 2019. The primary endpoint was the occurrence of SSI within 30 days post-operation; while secondary endpoints consisted of occurrence of superficial incisional SSI up to 30 days post-operation, occurrence of deep incisional SSI, occurrence of organ/space SSI, positive wound culture rate and strains detected in patients who underwent wound culture, and incidence of ADRs. Of the 597 patients who were enrolled, 587 were included in analysis; of these 587 patients, 294 patients and 293 patients were assigned to the olanexidine antisepsis group and the iodine antisepsis group, respectively. The number of cases of SSI within 30 days post-operation (the primary endpoint) was 39 in the conventional iodine antiseptic group versus 19 in the olanexidine antiseptic group, marking a 50% reduction in the incidence of SSI. Similarly, deep incisional SSIs occurred in 13 patients in the iodine antiseptic group versus 4 patients in the olanexidine antiseptic group, showing that the use of olanexidine significantly reduced the incidence of deep incisional SSIs. Furthermore, olanexidine did not demonstrate any new issues with safety.

2.3　Risks and benefits for study participants

In a non-clinical trial, aqueous olanexidine (the present investigational agent) demonstrated potent bactericidal activity against not only typical gram-positive and gram-negative bacteria but also MRSA, VRE, *Pseudomonas aeruginosa*, and strains said to demonstrate resistance to antiseptic agents, such as *Serratia sp.* and *Burkholderia cepacian*. Aqueous olanexidine demonstrates particularly potent bactericidal activity and fast action against gram-positive bacteria and could therefore help to inhibit SSI in surgical site skin antisepsis. Another benefit of aqueous olanexidine is that it can be used for patients who are allergic to alcohol. On the other hand, aqueous olanexidine poses a risk to patients who are allergic to olanexidine gluconate. However, we demonstrated the safety of aqueous olanexidine in the previously-cited clinical trial, and we will observe postoperative subjective and objective symptoms in study participants to detect ADRs early. Furthermore, to respond to ADRs quickly, in addition to listing major ADRs in the briefing form for obtaining consent, we will urge patients to contact their attending physician promptly in the event of worrisome symptoms and strive to minimize the risk to study participants. In addition, as previously stated, alcohol-based chlorhexidine (the control agent) is the first-line antiseptic agent in guidelines both in Japan and around the world, and it is used in daily clinical practice; therefore, its safety is considered to be ensured. Although there are still problems such as contact dermatitis, anaphylaxis, and flammability, these problems are considered to be rare if aqueous olanexidine is used correctly.

2.4　Route of administration, indication, and administration period

Aqueous olanexidine will be applied in moderation to the skin of the surgical site (operative field).

2.5　Observed regulations

The present study has been planned and will be conducted in compliance with the Declaration of Helsinki and Ethical Guidelines for Medical and Health Research Involving Human Subjects.

2.6　Trial participant population

Patients who meet the inclusion criteria (section 5.1) will be recruited at the above-listed centers.

**3　Objectives**

3.1　Background

Surgical site infection is one of the most common perioperative complications. In Japan, SSIs were observed in 7.2% of all surgeries from 2012 to 2015. In the United States, SSIs are reported to affect 300,000 to 500,000 patients each year and sometimes result in perioperative mortality. Furthermore, SSIs not only frequently prolong postoperative hospitalization but also require antimicrobial agents and bedside treatment, thereby potentially increasing healthcare costs. Thus, the prevention of SSIs is crucial not only for patients but also for all healthcare professionals involved in surgery and has therefore been the subject of much discussion.

The novel antiseptic agent 1.5% aqueous olanexidine is a novel biguanide antiseptic with olanexidine gluconate as its active ingredient. In a non-clinical trial, 1.5% aqueous olanexidine demonstrated potent bactericidal activity against not only typical gram-positive and gram-negative bacteria but also MRSA, VRE, *Pseudomonas aeruginosa*, and strains said to demonstrate resistance to antiseptic agents, such as *Serratia sp.* and *Burkholderia cepacian*. Aqueous olanexidine demonstrates particularly potent bactericidal activity and fast action against gram-positive bacteria and could therefore powerfully inhibit SSI in surgical site skin antisepsis and reduce rates of postoperative wound infection^1, 2^. The previously-cited randomized clinical trial proved that aqueous olanexidine is more effective for preventing SSIs than aqueous povidone-iodine, which has been used in Japan until now to prevent SSIs^3^. However, guidelines for preventing SSIs in gastrointestinal surgery and WHO guidelines recommend chlorhexidine-alcohol^4^. The two antiseptic agents have never been compared in a prospective trial, but such a comparison could serve as valuable evidence for surgical site antisepsis in the gastrointestinal region. One benefit of olanexidine gluconate versus chlorhexidine-alcohol is that it contains no alcohol and therefore poses no risk of the allergies and burns peculiar to alcohol; in addition, aqueous olanexidine is an applicator-type antiseptic and is therefore likely to help reduce healthcare costs. We have devised a randomized clinical trial to compare the efficacy of 1.0% alcohol-based chlorhexidine and 1.5% aqueous olanexidine for preventing SSIs.

3.2　Objectives

To investigate the SSI reduction effect of 1.5% aqueous olanexidine with that of 1% alcohol-based chlorhexidine on class II wounds in surgery in the gastrointestinal region.

**4　Trial design**

4.1 Endpoints

(1) Primary endpoint

Incidence of SSI within 30 days post-operation

(2) Secondary endpoints

Incidence of superficial incisional SSI within 30 days post-operation, incidence of deep incisional SSI, incidence of organ/space SSI, positive wound culture rate and strains detected in patients who underwent wound culture, incidence of ADRs (erythema, allergy symptoms, etc.), rate of reoperation due to SSI, medical economic effect indicators (data based on health insurance claims), duration of hospitalization

(3) Exploratory endpoints

None are established in the main analysis. However, secondary analysis may involve exploratory investigation of items such as risk factors for complications and risk factors in long-term outcomes.

(4) Safety endpoints

Rate of ADRs (erythema, allergy symptoms, etc.)

Surgical site infections are divided into superficial incisional SSIs, deep incisional SSIs, and organ/space SSIs. In accordance with United States Centers for Disease Control guidelines, SSIs are diagnosed according to the following criteria.

(i) Superficial incisional SSIs

Superficial incisional SSI must meet the following three criteria:

A) Infection occurs within 30 days post-operation

B) The infection involves only skin and subcutaneous tissue of the incision

C) At least one of the following:

a. Purulent drainage from the superficial incision.

b. Organism(s) isolated from an aseptically-obtained specimen from the superficial incision or subcutaneous tissue.

c. A superficial incision that is deliberately opened by a surgeon, and is culture-positive or has not been cultured. Also, at least one of the following signs or symptoms of infection must be present:

Pain, tenderness, localized swelling, erythema, or heat. This criterion is not met if the wound is culture-negative.

d. Diagnosis of superficial incisional SSI by a physician.

(ii) Deep incisional SSI

Deep incisional SSI must meet the following criteria.

A) Infection occurs within 30 days post-operation if no implant is left in place or within one year if an implant is left in place and the infection appears to be related to the operation.

B) The infection involves deep soft tissues (fascial and muscle layers) of the incision.

C) Patient has at least one of the following:

a. Purulent drainage from the deep incision but not from the organ/space component of the surgical site.

b. A deep incision spontaneously dehisces or is deliberately opened by a surgeon, and the wound is culture-positive or has not been cultured. Also, at least one of the following signs or symptoms must be present:

Fever (> 38°C), localized pain or tenderness. This criterion is not met if the wound is culture-negative.

c. An abscess or other evidence of infection involving the deep incision is found on direct examination, during reoperation, or by histopathologic or radiologic examination.

d. Diagnosis of a deep incisional SSI by a physician.

(iii) Organ/space SSI

An organ/space SSI can be in any area of the body other than the skin incision, fascial layer, or muscle layer that was opened or manipulated in the surgery. Specific sites are assigned to organ/space to further distinguish the infection site. Organ/space SSI must meet the following criteria.

A) Infection occurs within 30 days post-operation if no implant is left in place or within one year in an implant is left in place, and the infection appears to be related to the operation.

B) The infection involves any part of the anatomy that was opened or manipulated during the operative procedure (except the incision, fascial layer, or muscle layer)

C) Patient has at least one of the following:

a. Purulent drainage from a drain placed into the organ/space through a stab wound.

b. Organisms isolated from an aseptically obtained culture of fluid or tissue in the organ/space.

c. An abscess or evidence of infection involving the organ/space that is found on direct examination, during reoperation, or by histopathologic or radiologic examination.

d. Diagnosis of an organ/space SSI by a physician.

4.2 Trial methods

■Interventional study (clinical trial)

Invasiveness: ■Yes □No

Control group: ■Yes (Set in present task) □Yes (historical control) □No

Randomization: ■Yes　　　　　　　　□No

Blinding: ■Yes　　　　　 □No

Nature of study: □Exploratory trial ■Confirmatory trial

4.3 Bias

The following methods will be used to minimize bias in the trial.

1. Randomization: Participants will be assigned in a 1:1 ratio with the permuted block technique using a computer. Performance of laparoscopic surgery will be used as the assignment factor.
2. Double-blinding: Group assignments will be blinded both to the external Evaluation Committee for SSI Judgement and the study participants.

4.4 Investigational agents (investigational equipment, investigational treatments)

1) Olanedine® solution 1.5% OR antiseptic applicator 25 mL (Otsuka Pharmaceutical Factory, Inc.) Generic name: aqueous olanexidine

[Indication]

Surgical site (operative field) skin antisepsis

[Dosage and administration]

Aqueous olanexidine will be applied in moderation.

[Usage restrictions]

1. Careful administration (use carefully with the following patients)

(1) Patients with previous drug hypersensitivity

(2) Patients with previous allergic diseases (asthma, etc.) or a family history of the same

(3) Patients with previous hypersensitivity to chlorhexidine preparation

2. Important basic precautions

When using aqueous olanexidine, thoroughly confirm previous hypersensitivity to its components and predisposition to drug hypersensitivity.

[Major ADRs]

In a phase III clinical trial conducted at 16 Japanese centers with patients scheduled to undergo laparoscopic gastrointestinal surgery, a total of 3 ADRs were observed in 3 of 52 patients (5.8%) in the safety analysis set. These ADRs consisted of one incidence each of application site dermatitis, application site erythema, and application site pruritus in 1 participant each (1.9%)^5^.

2) Chlorhexidine gluconate ethanol antiseptic solution 1% Toho Generic name: Chlorhexidine gluconate

[Indication]

Hand/skin antisepsis

[Dosage and administration]

Apply a moderate amount to hands/skin after washing several times a day to disinfect them.

[Usage restrictions]

1. Careful administration (use carefully with the following patients)

(1) Patients with previous drug hypersensitivity

(2) Patients with previous allergic diseases (asthma, etc.) or a family history of the same

2. Important basic precautions

1. Prior to use, due to anticipated reactions such as shock and anaphylaxis, thorough interviews should be conducted with patients regarding past hypersensitivity to chlorhexidine preparations and predisposition to drug hypersensitivity.

2. Use the solution as is without diluting.

3. Do not use for obstetric/gynecological purposes (antisepsis of the vagina/vulva, etc.) or urological purposes (antisepsis of the bladder/external genitalia, etc.).

4. Be careful to keep it out of the eyes. If it gets in the eyes, immediately wash them thoroughly.

5. If using over a wide area or for a long period, be careful to avoid inhaling vapor. (Inhalation of large volumes of ethanol vapor or repeated inhalation can irritate mucous membranes and cause headaches.)

[Major ADRs]

Shock (< 0.01%), anaphylaxis (frequency unknown)

As shock or anaphylaxis may occur, observe the patient thoroughly. In the event of symptoms such as hypotension, urticaria, or dyspnea, discontinue immediately and perform suitable treatment.

[Other notes]

Due to the presence of ethanol, keep away from flames.

*Although the package insert does not give specific numbers, a PMDA report has cited 9 cases of ethanol catching fire from 2004 to 2014. Many of these cases involved an electric scalpel being used before the alcohol had dried or a lack of knowledge of drugs. All persons involved in the present trial will be thoroughly instructed regarding the flammability of ethanol. Also, to reduce this risk as much as possible, incisions will be made no fewer than 3 minutes following the conclusion of skin antisepsis. As the preparation is reported to dry 41 seconds after it is applied to the skin, the prescribed 3-minute interval is considered sufficient.

4.5 Study period

Date of IRB approval – 12/31/2025

4.6 Suspension/discontinuation of trial

(1) Trial suspension/discontinuation criteria for individual participants

In the event of any of the following after enrollment, the principal investigator or co-investigator will discontinue the trial for that participant and promptly inform the secretariat of the date of and reason for discontinuation.

1. The participant asks to withdraw.
2. The participant asks to withdraw due to an adverse event.
3. The attending physician, principal investigator, or co-investigator deems continuation of the trial difficult due to an adverse event.
4. The trial itself is discontinued.

(2) Trial suspension/discontinuation criteria for part or all of the trial itself

In the event that the trial is discontinued or suspended or the trial protocol must undergo major revision while the trial is in progress due to the following reasons, the principal investigator or co-investigator will promptly inform the School of Medicine Dean in writing.

1. Participants are difficult to recruit, or the target sample size is deemed difficult to reach
2. The IRB demands revisions to the trial protocol that are deemed difficult to accept

4.7 Management of investigational agents (investigational equipment, investigational treatments)

The investigational agent and the control agent will be managed by the operating room pharmaceutical department. Patients will not personally manage the investigational agents in the present study.

4.8 Protection of participant privacy

(1) Own center’s samples/data □N/A (none used)

Anonymization: ■Yes □No

(If yes) Performed by: ■ Own center (in principle) □Other ( )

Anonymization technique: ■Linkable □Non-linkable

(If anonymization is linkable) Linking table managed by: ■Own center (in principle)□Other ( )

*Specific methods of anonymization and management of linking table (if no anonymization, explain why):

We will collect data which is based on medical records and test records; and we will ensure that names, medical record IDs, and all other information that can be used to identify individuals is deleted. Linkable primary anonymization numbers will then be assigned, and participants’ information will be securely stored in a locked bookshelf at the Department of Surgery (General/Gastrointestinal), Keio University School of Medicine by the personal information manager.

(2) Other centers’ sample/data: □N/A (none used)

Anonymization: ■Yes □No

(If yes) Performed at: ■The center (in principle) □Other ( )

Anonymization technique: ■Linkable □Non-linkable

(If anonymization is linkable) Linking table managed at: ■The center (in principle)□Other ( )

4.9 Raw data

Raw data is defined as the following: basic patient information included in medical records (age, sex, operative procedure, etc.), clinical data established as the primary endpoint and secondary endpoints (SSI rate within 30 days post-operation, superficial incisional SSI rate within 30 days post-operation, deep incisional SSI rate, organ/space SSI rate, positive wound culture rate and strains detected in patients who underwent wound culture, clinical information to assess ADR rates), laboratory data (wound culture results, etc.), and DPC data related to medical records. Clinical-pathological factors, operative findings, operative outcomes, and long-term outcomes will also be collected as raw data.

**5　Participant inclusion/exclusion/withdrawal criteria**

5.1　Inclusion criteria

Patients who meet all of the following conditions will be included.

1. Patients undergoing elective surgery under general anesthesia in the gastrointestinal region (esophagus, stomach, duodenum, small intestine, large intestine, liver, biliary tract, pancreas) with class II wounds
2. Age ≥ 18 years at the time of consent to participate in the trial
3. Provision of written consent to participate in the trial

5.2　Exclusion criteria

Patients to whom any of the following conditions apply will be excluded.

1)　Patients with a history of allergy to aqueous olanexidine, alcohol-based chlorhexidine, or any other alcohol

2)　Patients unable to undergo 30-day postoperative follow-up

3)　Patients with active infections

4)　Patients who use antimicrobials the day before surgery (except for preoperative prophylactic antimicrobials in colorectal surgery)

5)　Emergency surgery or surgery requiring antisepsis of mucosal surfaces or wounds

6)　Patients with a history of asthma

7)　Patients deemed by a physician to be ineligible to participate in the trial safely for any other reason

5.3　Withdrawal criteria

See 4.6(1): Suspension/discontinuation of trial

**6　Investigational treatments**

6.1　Details of investigational treatments

The present trial is a prospective randomized trial which assessed a primary endpoint and secondary endpoints with participants divided into the two groups described below. The trial will last for 30 days following surgery, during which time an attending physician will observe wounds daily. After discharge, in principle, participants will visit the hospital to undergo examination at least once within 30 days post-operation; the outpatient physician will observe wounds in the same manner as the attending physician. Regular hospital visits are not required at 30 days post-operation; however, if the wound presents with abnormal findings (redness, pain, heat sensation, pus, other new subjective or objective findings), the patient will visit the hospital regularly. The evaluator who are in the External Evaluation Committee for SSI Judgement, who will be blinded, will determine the presence of SSI based on the case report form written by the attending physician.

Group A: Operative field antisepsis immediately prior to operation will consist of antisepsis of the front and back of the trunk with 1.5% aqueous olanexidine (trade name: Olanedine® solution 1.5% OR antiseptic applicator 25 mL). On the cranial and caudal sides, antisepsis will be performed to the height of the nipples (up to the neck in the case of surgery for esophageal cancer) and down to the upper thighs, respectively. Additional antisepsis during surgery will also be performed with 1.5% aqueous olanexidine. The incision will be performed no less than 3 minutes following the conclusion of skin antisepsis.

Group B: Operative field antisepsis immediately prior to operation will consist of antisepsis of the front and back of the trunk with 1% alcohol-based chlorhexidine (trade name: Chlorhexidine gluconate ethanol antiseptic solution 1%). On the cranial and caudal sides, antisepsis will be performed to the height of the nipples (up to the neck in the case of surgery for esophageal cancer) and down to the upper thighs, respectively. The agent will be applied three times concentrically from inside to outside with a cotton swab, sponge, or brush. Additional antisepsis during surgery will also be performed with 1% alcohol-based chlorhexidine. The incision will be performed no less than 3 minutes following the conclusion of skin antisepsis.

In accordance with Global Guidelines for the Prevention of Surgical Site Infection, other perioperative management prescriptions are established as listed below.

1. Administer antimicrobials prior to incision
2. Use absorbable sutures to close wounds. Antibacterial sutures are preferable
3. The use of a wound retractor in laparotomy is irrelevant but preferable
4. Saline irrigation of wounds prior to closure is preferable but not required
5. The use versus non-use of immunosuppressants is irrelevant
6. Changing versus no change of gloves prior to wound closure is irrelevant
7. Changing versus no change of surgical implements prior to wound closure is irrelevant

6.2 Combination therapy

(1) Therapies permitted prior to or during the clinical trial (including therapy for emergencies)

All therapies other than those which use the investigational agent are permitted.

(2) Therapies prohibited prior to or during the clinical trial

Due to potential effects on trial results, the use of antimicrobials the day before surgery is prohibited. (See 5.2: Exclusion criteria)

6.3 Compliance

The attending physician and ward nurse will observe wounds every day post-operation. After discharge, in principle, participants will visit the hospital to undergo examination at least once within 30 days post-operation; the outpatient physician and outpatient nurse will observe wounds in the same manner as the attending physician.

**7 Efficacy assessment**

7.1 Efficacy assessment indicators

See 4.1: Endpoints

7.2 Assessment, recording, and analysis of efficacy assessment indicators

Case report forms will be completed as appropriate. The following items will be abstracted from case report forms: date of surgery, name of operation, wound classification, ASA, performance of endoscopy, wound closure method, prophylactic antimicrobials, repeated administration of antimicrobials due to surgery lasting ≥ 3 hours, antimicrobial sutures used for wound closure, intrabdominal irrigation volume, wound irrigation volume, presence of diabetes, presence of undernutrition, administration of steroids for ≥ 7 days, SSI assessment criteria items, and occurrence of AEs. The following items will also be abstracted: re-operation due to SSI, medical economic effect indicators (data based on health insurance claims), duration of hospitalization, clinical-pathological factors, operative findings, surgical outcomes, and prognosis. Performance of endoscopy is assessed as “no” if the operation began as an endoscopy but was changed to thoracotomy or laparotomy. In esophageal surgery, performance of endoscopy is assessed as “no” if a thoracic approach involved thoracotomy or an abdominal approach involved laparotomy. Hand-assisted laparoscopic surgery is included in laparoscopic surgery.

(1) Assessment methods

See 4.1 (i) (ii) (iii)

(2) Analysis methods

After all cases have been accumulated, we will examine the presence of statistical differences alongside statisticians at the Clinical & Translational Research Center using the χ^2^ test or Fisher’s exact test, with p < 0.05 considered significant.

**8 Safety assessment**

8.1 Safety assessment indicators

See 4.1(4): Safety endpoints

8.2 Assessment, recording, and analysis of safety assessment indicators

Case report forms will be completed as appropriate.

(1) Assessment methods

See 4.1(4)

(2) Analysis methods

As in 7.2(2).

8.3　Adverse events

(1)　Definition of AE

The definition of an AE in ICH (International Conference on Harmonisation of Technical Requirements for Registration of Pharmaceuticals for Human Use)-GCP (Good Clinical Practice) is shown below.

“An adverse event is any untoward medical occurrence in a patient or clinical investigation subject administered a pharmaceutical (investigational) product and which does not necessarily have a causal relationship with this treatment. An AE can therefore be any unfavourable and unintended sign (including an abnormal laboratory finding), symptom, or disease temporally associated with the use of a medicinal (investigational) product, whether or not related to the medicinal (investigational) product”.

(2)　Definition of serious adverse event (SAE)

Serious adverse events are defined in ICH-GCP as shown below.

| Serious Adverse Events (SAEs) |
| --- |
| Results in death |
| Is life-threatening |
| Requires inpatient hospitalization or prolongation of existing hospitalization |
| Results in persistent or significant disability/incapacity |
| Is a congenital anomaly/birth defect |
| Any other serious medical event |

(3) Procedure for assembling, recording, and reporting AEs/SAEs

i)　Events to be reported

1. Events resulting in death

・Deaths occurring within 30 days post-operation that have a causal relationship with the investigational therapy. Evident death from the original disease is not counted as intraoperative death.

1. Life-threatening events

・CTCAE Grade 3 AEs occurring within 48 hours after administration of the investigational agent and which have a causal relationship with the investigational treatment. Evident complications of surgery are not counted here.

1. Events requiring inpatient hospitalization or prolongation of existing hospitalization

However, events without a causal relationship with the investigational treatment are excluded.

1. Events resulting in persistent or significant disability/incapacity

・Aplastic anemia, myelodysplastic syndromes, secondary cancer, etc. However, events without a causal relationship with the investigational treatment are excluded.

1. Events that are congenital anomalies/birth defects. However, events without a causal relationship with the investigational treatment are excluded.
2. Any other serious medical events. However, events without a causal relationship with the investigational treatment are excluded.

Any events that do not fit 2) through 5) above but are considered medically serious.

Responses to SAEs are described below. The reporting period will last only for the duration of the trial period.

| Causal relationship | Predictability | Seriousness | Reporting |
| --- | --- | --- | --- |
| Yes | Predictable (listed in package insert) | Death | Emergency report (promptly) |
|  |  | Life-threatening (within 48 hours after investigational agent administration) | Normal report (within 15 days) |
|  | Unpredictable (not listed in package insert) | Death | Emergency report (promptly) |
|  |  | Life-threatening (within 48 hours after investigational agent administration) |  |
| No | Report not required | | |

ii)　Reporting methods (emergency report, other)

Reports are made to the head of the study center according to the designated procedure.

iii)　Report destination (study center head, other study center)

Reports will be made to the head of the study center according to the designated procedure. Applicable AEs will be made known to the clinical study center. The physician in charge will report to the investigational agent distributor (person in charge of drug information) if they deem it necessary. In the event of an unpredictable SAE with a causal relationship to the investigational agent, the physician in charge at the study center where the SAE occurred will file an ADR report with the Minister of Health, Labour and Welfare based on the Act on Securing Quality, Efficacy and Safety of Products Including Pharmaceuticals and Medical Devices.

1. Olanedine® solution 1.5% OR antiseptic applicator 25 mL: Otsuka Pharmaceutical Factory, Inc.

 Address: 2-9 Kanda-Tsukasamachi, Chiyoda-ku, Tokyo, 101-8535, Japan

TEL: 03-6717-1400 (Main)

　　　　FAX: 03-6717-1499

1. Chlorhexidine gluconate ethanol antiseptic solution 1% Toho: Yoshida Pharmaceutical Co., Ltd.

 Address: Chuo 5-1-10, Nakano-ku, Tokyo, 164-0011, Japan

TEL: 03-3381-7291 (Main)

　　　　FAX: 03-3381-7244

8.4 Follow-up of participants after presentation of AEs

(1) Compensation for health damage (yes/no)

The present study appropriately uses both investigational agents as surgical site (operative field) skin antiseptics within the scope of application in the package inserts; therefore, the use of these agents themselves is within the scope of everyday treatment. In addition, the present study does not involve any invasive tests or blood collection unique to the study during the trial period. However, the ADRs listed in the package inserts may occur. If participants experience these ADRs or any other AEs, participants will be given the best treatment available covered by normal health insurance.

**9 Statistical analysis**

9.1 Primary analysis

The primary efficacy endpoint is the incidence of SSI within 30 days post-operation. The primary objective of the present trial is to examine the superiority of aqueous olanexidine (investigational treatment group) versus alcohol-based chlorhexidine (standard treatment group) in preventing SSI in patients undergoing surgery in the gastrointestinal region with class II wounds. Estimates are calculated for the adjusted risk ratio and its two-sided 95% confidence interval (CI) for the rate of SSI in the investigational treatment group versus the standard treatment group. The adjustment factor is the assignment factor (performance of laparoscopy). In the primary analysis, the Mantel-Haenszel test, with the assignment factor as the stratification factor, will be performed with the null hypothesis that the rate of SSI is equal in both groups. The test statistic is based on the log Mantel-Haenszel risk ratio estimator in Greenland & Robins (Biometrics 1985;41(1):55–68). The level of statistical significance is set at 5% (two-tailed). Frequencies and ratios will be tabulated for both groups, and two-sided 95% CIs will be calculated for ratios.

9.2 Secondary analysis

The secondary endpoints are the following: incidence of superficial incisional SSIs up to 30 days post-operation, incidence of deep incisional SSI, incidence of organ/space SSI, positive wound culture rate and strains detected in patients who underwent wound culture, incidence of ADRs (erythema, allergy symptoms), rate of reoperation due to SSI, medical economic effect indicators, and duration of hospitalization. Except for medical economic effect indicators, all of these will be analyzed with the same methods as in the primary analysis. Details are given separately in the statistical analysis plan. For analysis of medical economic effect indicators, cost-effective analysis alongside clinical trials will be performed. Incremental cost-effectiveness ratio (yen/event) per case of SSI in the intervention group and the control group will be calculated based on incidence of SSI within 30 days post-operation (the primary endpoint) and data based on health insurance claims (collected as a secondary endpoint). In accordance with the policies of the International Society for Pharmacoeconomics and Outcomes Research, uncertainty assessment and sensitivity analysis will be performed for sampling, parameters, and imputation, and the robustness of the results will be confirmed. If the null hypothesis in the primary analysis is not rejected, cost-minimization analysis will be performed based on the hypothesis that olanexidine and chlorhexidine are of equivalent efficacy, and the difference in cost between the two products will be assessed^6^.

9.2 Sample size

Target sample size: 700

Basis for sample size estimation: In a meta-analysis, the rate of SSI in class II wounds disinfected with alcohol-based chlorhexidine was roughly 13.0%. In a previous RCT, the incidence of SSI with olanexidine was 6.5%; therefore, the SSI incidence in the present study is also predicted to be roughly 6.5%. If statistical power is set at 80%, 326 participants are needed per group. Due to the need for both groups to be equal in size and a presumed dropout rate of roughly 10%, the target sample size was calculated as 350 per group, thus a total of 700^7^.

9.3 Level of statistical significance

Set at 5% (two-tailed).

9.4 Trial discontinuation criteria

🡪See 4.6: Suspension/discontinuation of trial

9.5 Handling of trial data

If missing values emerge, that case will not be used in comparisons regarding the item in question. If outliers emerge, the data will be used unless there is an obvious miscalculation.

9.6 Deviation from statistical analysis plan

Publication of results for analysis which deviates from the statistical analysis plan will be accompanied with the following clear explanation: “These are results for analysis which deviates from the initial statistical analysis plan”. This explanation will be given at times such as manuscript publication and academic society presentation.

9.7 Analysis sets

(1) Selection of participants for analysis

□Intention-to-treat (ITT)

■Full analysis set (FAS)

■Per protocol set (PPS)

(2) Definition and determination of evaluable cases

Among all cases, those evidently in conflict with the inclusion/exclusion criteria and those who do not receive the investigational agent are excluded.

(3) Other considerations

None in particular

**10 Source data verification for raw data and source documents**

The principal investigator and the clinical trial centers will provide raw data/source documents for source data verification for the following occasions: clinical trial monitoring, auditing, review by the IRB or other bodies, and inspection by authorities.

**11 Monitoring and auditing**

11.1 Monitoring

The present trial involves only slight invasiveness and therefore does not require monitoring. However, while the trial will be conducted safely according to the protocol, central monitoring and on-site monitoring will be performed to ensure that the data is collected accurately.

The main center will undergo on-site monitoring for the first 10 cases following the start of the trial. Other participating centers will conduct self-inspection for the first five cases following the start of the trial according to the same format and will submit these self-inspections to the main facility. Self-inspections will be performed at every center for researchers who have not enrolled patients (obtained consent). Monitoring will be changed to central monitoring if the secretariat and monitor determine based on the results of on-site monitoring that doing so would not interfere with continuation of the trial. Central monitoring will be performed once annually with roughly 5% of enrolled patients. While monitoring with 5% of the established sample size is considered sufficient, central monitoring will be extended to more cases as appropriate if the secretariat and monitor deem the number of cases to be insufficient.

Monitoring will be performed primarily by the secretariat and the monitor with the support of the Monitoring Unit in the Division of Clinical Research Support, Clinical & Translational Research Center, Keio University Hospital. On-site monitoring will consist of tasks including but not limited to the following: confirmation of IRB audit-related processes, including cross-checking with the facility’s source materials, and of original copies of related materials; confirmation of processes for obtaining consent; confirmation of storage of original copies of consent forms and source materials; confirmation of eligibility; maintenance of assignment and blinding; confirmation of occurrence of SAEs and the process for reporting them; confirmation of adherence to the trial protocol; and confirmation of consistency between source materials and case report forms. Central monitoring will consist primarily of the following: confirmation of ethical review-related documents, numbers of individuals who have provided consent, numbers of participants (enrollment numbers), numbers of participants who have withdrawn from or dropped out and their reasons for doing so, informed consent, safety information, participant eligibility (inclusion/exclusion criteria), and accuracy of case enrollment/assignment procedures; and compliance with regulations and study protocol.

11.2 Auditing

The principal investigator and secretariat will examine the need for auditing regarding matters including but not limited to reported SAEs and deviations from trial protocol discovered through monitoring and will conduct audits as necessary.

**12 Ethical considerations**

All researchers involved with the present trial will conduct the trial in accordance with the Declaration of Helsinki (October 2013, Fortaleza revision) and Ethical Guidelines for Medical and Health Research involving Human Subjects (Ministry of Education, Culture, Sports, Science and Technology; Ministry of Health, Labour and Welfare; announced 12/22/2014).

12.1 Informed consent

The principal investigator or co-investigator will obtain informed consent using a form approved by an in-house ethical review board (IRB/IEC) to thoroughly explain the following: the objective of the trial, AEs, the trial period, the free nature of participation, the absence of disadvantage in treatment if consent is not provided, and the freedom to withdraw consent at any time. Patients will be given enough time to ask questions and decide whether they wish to participate in the trial.

After confirming that the patient has thoroughly understood the nature of the trial, the investigator will obtain the patient’s consent to participate in the trial of their own free will using a consent form. The original consent form will be stored with the patient’s medical records, one copy will be given to the patient, and another copy will be stored at the trial center.

12.1.1 Obtainment of consent from proxies

■No (no plan to obtain consent from proxies)

□Yes: Explanation and consent provided in writing

□Yes: Explanation and consent provided orally, with a written record

□In principle, but trial will be conducted without IC if difficult to obtain

12.2 Reports to study center head

Reports will be made annually based on center criteria. Reports will also be made in regard to trial suspension, discontinuation, and conclusion based on center criteria.

12.3 Disclosure of study-related information

Information related to the present study will be published on the website of the Department of Surgery (General/Gastrointestinal), Keio University School of Medicine.

12.4 Response to questions from participants and related persons

Practical investigator: Masashi Takeuchi

Assistant Professor, Department of Surgery (General/Gastrointestinal), Keio University School of Medicine

Address: 35 Shinanomachi, Shinjuku-ku, Tokyo, 160-8582, Japan

TEL：03-5363-3802 FAX：03-3355-4707

12.5 Conflicts of interest

The present study will be conducted with research grants from Novartis Pharma K.K and Johnson & Johnson K.K. In regard to researchers’ conflicts of interest in the present study, the study will be conducted with the conflict of interest management committee at each center having judged that the study can be conducted fairly.

12.6 Handling of participants following conclusion of trial

Following the conclusion of the trial, participants will continue to undergo regular examination.

12.7 Handling of important findings related to genetic characteristics, etc.

Not applicable in the present study.

**13 Handling of data/samples and preservation of records**

See 4.8: Protection of participant privacy

13.1 Potential use of data/samples in other future studies and provision to other centers

Data obtained in the present trial may be used among co-investigators in secondary analysis. Secondary analysis using only data obtained in the trial will not require application for an ethical review; however, if further data is to be collected, an application for an ethical review will be filed with the School of Medicine IRB.

**14 Economic burden on participants and measures other than insurance**

1. Economic burden on participants associated with trial participation

The present trial will be conducted within the scope of treatment covered by health insurance and therefore does not increase costs borne by patients.

(2) Issuance of reimbursements to participants

No particular reimbursements will be issued.

(3) Measures other than insurance for compensation for health damage

The present study appropriately uses both investigational agents as surgical site (operative field) skin antiseptics within the scope of application in the package inserts; therefore, the use of these agents themselves is within the scope of everyday treatment. In addition, the present study does not involve any invasive tests or blood collection unique to the study during the trial period. However, the ADRs listed in the package inserts may occur. If participants experience these ADRs or any other AEs, participants will be given the best treatment available covered by normal health insurance. Serious ADRs resulting from use of the investigational agents will be covered by the Adverse Drug Reaction Relief System.

The present study will measure items measured in everyday treatment; therefore, participation in the study does not involve additional invasiveness or AEs.

**15 Arrangements regarding publication of trial results**

After the trial is concluded, its results will be suitably presented at academic conferences in Japan and elsewhere and published in English-language journals.

The final study report will be the English-language journal article once it is published.

**16 Supplement**

Olanedine® solution 1.5% OR antiseptic applicator 25 mL (Otsuka Pharmaceutical Factory, Inc.)

**17 References**

1. Sakagami Y, et al：Electron-microscopic study of the bactericidal effect of OPB-2045, a new disinfectant produced from biguanide group compounds, against methicillin-resistant Staphylococcus aureus J Pharm Pharmacol 2000；52(12)：1547-1552
2. Inoue Y, et al：Novel antiseptic compound OPB-2045G shows potent bactericidal activity against methicillin resistant Staphylococcus aureus and vancomycin resistant Enterococcus both in vitro and in vivo: a pilot study in animals J Med Microbiol. 2015；64(1)：32-36
3. Obara H, Takeuchi M, Kawakubo H, Shinoda M, Okabayashi K, Hayashi K, Sekimoto Y, Maeda Y, Kondo T, Sato Y, Kitagawa Y. Aqueous olanexidine versus aqueous povidone-iodine for surgical skin antisepsis on the incidence of surgical site infections after clean-contaminated surgery: a multicentre, prospective, blinded-endpoint, randomised controlled trial. Lancet infectious disease 2020 S1473-3099(20)30225-5. doi: 10.1016/S1473-3099(20)30225-5.
4. World Health Organization: Global Guidelines for the Prevention of Surgical Site Infection 2016.
5. Investigational agent package inserts
6. Ramsey SD, Willke RJ, Glick H, Reed SD, Augustovski F, Jonsson B, Briggs A, Sullivan SD. Cost-effectiveness analysis alongside clinical trials II-An ISPOR Good Research Practices Task Force report. Value Health. 2015 Mar;18(2):161-72. doi: 10.1016/j.jval.2015.02.001. PMID: 25773551.
7. National Institute of Health Research Unit on Global Surgery. Alcoholic chlorhexidine skin preparation or triclosan-coated sutures to reduce surgical site infection: a systematic review and meta-analysis of high-quality randomised controlled trials. Lancet Infect Dis. 2022 Aug;22(8):1242-1251. doi: 10.1016/S1473-3099(22)00133-5. Epub 2022 May 26. Erratum in: Lancet Infect Dis. 2022 Jun 1;: PMID: 35644158.
