# Supplementary material for: Efficacy of aqueous olanexidine compared with alcohol-based chlorhexidine for surgical skin antisepsis regarding the incidence of surgical-site infections in clean-contaminated surgery: a randomized superiority trial
Source: Br J Surg. 2025 Apr 1;112(4):znaf065. doi: 10.1093/bjs/znaf065 (PMC12475902; doi:10.1093/bjs/znaf065)
Supplement: znaf065_Supplementary_Data [file znaf065_supplementary_data.zip › Supplementary_Material.docx]

**Title: The efficacy of aqueous olanexidine compared to alcohol-based chlorhexidine for surgical skin antisepsis on incidence of surgical site infections in clean-contaminated surgery: a randomised superiority trial**

Masashi Takeuchi, M.D., Ph.D.^1^; Hideaki Obara, M.D., Ph.D.^1^; Tasuku Furube, M.D.^1^; Hirofumi Kawakubo, M.D., Ph.D.^1^; Minoru Kitago, M.D., Ph.D.^1^; Koji Okabayashi, M.D.^1^; Hiroto Fujisaki, M.D., Ph.D.^2^; Junya Aoyama, M.D., Ph.D.^3^; Yosuke Morimoto, M.D., Ph.D.^3^, Ryusuke Amemiya, M.D., Ph.D.^4^; Junichi Sano, M.D. , Ph.D.^5^; Jumpei Nakadai, M.D., Ph.D.^5^; Rei Goto, M.D., Ph.D.^6^; Yasunori Sato, Ph.D.^7^; and Yuko Kitagawa, M.D., Ph.D.^1^

^1^Department of Surgery, Keio University School of Medicine, 35 Shinanomachi, Shinjuku-ku, Tokyo 160-8582, Japan

^2^Department of Surgery, Hiratsuka City Hospital, 1-19-1 Minamihara, Hiratsuka, Kanagawa 254-0065, Japan

^3^Department of Surgery, Saiseikai Yokohamashi Tobu Hospital, 3-6-1 Shimosueyoshi, Tsurumi-ku, Yokohama, Kanagawa 230-8765, Japan

^4^Department of Surgery, Kawasaki Municipal Hospital, 12-1 Shinkawadori, Kawasaki-ku, Kawasaki, Kanagawa 210-0013, Japan

^5^Department of Gastrointestinal Surgery, Saitama City Hospital, 2460 Mimuro, Midori-ku, Saitama, Saitama 336-8522, Japan

^6^Graduate School of Business Administration, Keio University, 4-1-1 Hiyoshi, Yokohama 223-8526, Japan

^7^Department of Biostatistics, Keio University School of Medicine, 35 Shinanomachi, Shinjuku-ku, Tokyo 160-8582, Japan

**Corresponding author:**

Hideaki Obara, M.D., Ph.D.

Department of Surgery, Keio University School of Medicine, 35 Shinanomachi, Shinjuku-ku, Tokyo 160-8582, Japan

E-mail: obara.z3@keio.jp; Tel: +81-3-3353-1211; Fax: +81-3-3755-4707

**Supplementary Materials - Index**

| **Supplementary Figures and Tables** |  |
| --- | --- |
| Table S1 Types of surgical procedures in the full analysis set | *Page3* |
| Table S2 Primary and secondary outcomes according to the intervention group in the per-protocol set | *Page4* |
| Table S3 Primary and secondary outcomes according to the intervention group in the intention to treat set | *Page5* |
| Table S4 Positive bacterial cultures and bacterial strains | *page 6* |

**Supplementary Tables**

**Table S1** Types of surgical procedures in the full analysis set

|  | **Olanexidine (n = 347)** | **Chlorhexidine-alcohol (n = 345)** |
| --- | --- | --- |
| Upper gastrointestinal | 111 (31·99%) | 89 (25·80%) |
| Esophagus | 30 (8·65%) | 26 (7·54%) |
| Stomach | 81 (23·34%) | 63 (18·26%) |
| Lower gastrointestinal | 73 (21·04%) | 71 (20·58%) |
| Appendix | 9 (2·59%) | 9 (2·61%) |
| Colon | 45 (12·97%) | 46 (13·33%) |
| Rectum | 19 (5·48%) | 16 (4·64%) |
| Hepato-biliary-pancreatic | 156 (44·96%) | 179 (51·88%) |
| Gall bladder | 95 (27·38%) | 107 (31·01%) |
| Liver | 36 (10·37%) | 37 (10·72%) |
| Pancreas | 25 (7·20%) | 35 (10·14%) |
| Other | 7 (2·02%) | 6 (1·74%) |

**Table S2** Primary endpoint and secondary outcomes according to the intervention group in the per-protocol set

|  | **Olanexidine (n = 343)** | **Chlorhexidine-alcohol (n = 341)** | **Adjusted risk difference (95% CI)** | **Adjusted risk ratio (95% CI)** | **P** |
| --- | --- | --- | --- | --- | --- |
| Primary endpoint |  |  |  |  |  |
| SSI - no (%) | 42 (12·24) | 47 (13·78) | −0·014 (−0·063 - 0·035) | 0·899 (0·615 - 1·313) | 0·550 |
|  |  |  |  |  |  |
| Secondary outcomes (Type of SSI) |  |  |  |  |  |
| Superficial incisional | 17 (4·96) | 19 (5·57) | −0·006 (−0·039 - 0·028) | 0·896 (0·475 - 1·693) |  |
| Deep incisional | 3 (0·87) | 5 (1·47) | −0·006 (−0·022 - 0·010) | 0·603 (0·146 - 2·490) |  |
| Organ-space | 22 (6·41) | 23 (6·74) | −0·002 (−0·039 - 0·034) | 0·966 (0·555 - 1·68) |  |
|  |  |  |  |  |  |
| Secondary outcomes (Adverse skin reaction) |  |  |  |  |  |
| All | 2 (0·58) | 3 (0·88) | −0·003 (−0·016 - 0·01) | 0·669 (0·114 - 3·931) |  |
| Erythema | 0 (0) | 3 (0·88) | −0·009 (−0·019 - 0·001) | 0·141 (0·007 - 2·718) * |  |
| Pruritus | 1(0·29) | 0 (0) | 0·003 (−0·003 - 0·009) | 3·162 (0·133 - 75·2) * |  |
| Dermatitis | 2 (0·58) | 0 (0) | 0·006 (−0·002 - 0·014) | 3·061 (0·323 - 29·05) * |  |
|  |  | − |  |  |  |
| Secondary outcomes  Reoperation caused by SSI | 2 (0·59) | 4 (1·17) | −0·006 (−0·020 - 0·008) | 0·497 (0·092 - 2·692) |  |

SSI, surgical site infection· * the use of continuity correction of 0·5 for zero-event outcome·

**Table S3** Primary endpoint and secondary outcomes according to the intervention group in the intention to treat set

|  | **Olanexidine (n = 350)** | **Chlorhexidine-alcohol (n = 350)** | **Adjusted risk difference (95% CI)** | **Adjusted risk ratio (95% CI)** | **P** |
| --- | --- | --- | --- | --- | --- |
| Primary endpoint |  |  |  |  |  |
| SSI - no (%) | 43 (12·29) | 47 (13·43) | −0·011 (−0·061 - 0·039) | 0·904 (0·574 - 1·423) | 0·664 |
|  |  |  |  |  |  |
| Secondary outcomes (Type of SSI) |  |  |  |  |  |
| Superficial incisional | 17 (4·86) | 19 (5·43) | -0·006 (−0·038 - 0·027) | 0·892 (0·455 - 1·749) |  |
| Deep incisional | 3 (0·86) | 5 (1·43) | −0·006 (−0·025 - 0·013) | 0·599 (0·142 - 2·527) |  |
| Organ-space | 23 (6·57) | 23 (6·57) | 0·001 (−0·036 - 0·037) | 1.008 (0·548 - 1·854) |  |
|  |  |  |  |  |  |
| Secondary outcomes (Adverse skin reaction) |  |  |  |  |  |
| All | 2 (0·57) | 3 (0·87) | −0·028 (−0·015 - 0·010) | 0·668 (0·112 - 4·003) |  |
| Erythema | 0 (0) | 3 (0·86) | −0·009 (−0·018 - 0·001) | 0·141 (0·007 - 2·743) * |  |
| Pruritus | 1(0·29) | 0 (0) | 0·003 (−0·003 - 0·009) | 3·160 (0·125 - 80·0) * |  |
| Dermatitis | 2 (0·57) | 0 (0) | 0·006 (−0·002 - 0·014) | 3·078 (0·316 - 29·9) * |  |
|  |  | − |  |  |  |
| Secondary outcomes  Reoperation caused by SSI | 2 (0·58) | 4 (1·16) | −0·057 (−0·019 - 0·008) | 0·495 (0·090 - 2·723) |  |

SSI, surgical site infection· * the use of continuity correction of 0·5 for zero-event outcome·

**Table S4** Positive bacterial cultures and bacterial strains

|  | **Olanexidine (n = 347)** | **Chlorhexidine-alcohol (n = 345)** |
| --- | --- | --- |
| Performing bacterial test (no·) | 49 | 41 |
| Identification of pathogen | 35 (71·43%) | 34 (82·93%) |
| Pathogens |  |  |
| *Enterococcus* sp. | 12 (24·49%) | 12 (29·27%) |
| *Enterobacter* sp. | 9 (18·37%) | 8 (19·51%) |
| *Bacteroides* sp. | 1 (2·04%) | 1 (2·44%) |
| *Staphylococcus aureus* | 1(2·04%) | 2 (4·88%) |
| *Staphylococcus epidermidis* | 2 (4·08%) | 1 (2·44%) |
| *Staphylococcus* sp. | 2 (4·08%) | 3 (7·32%) |
| *Streptococcus* sp. | 5 (10·20%) | 2 (4·88%) |
| *Pseudomonas aeruginosa* | 9 (18·37%) | 6 (14·63%) |
| *Corynebacterium* sp. | 2 (4·08%) | 1 (2·44%) |
| *Klebsiella* sp. | 3 (6·12%) | 6 (14·63%) |
| *Escherichia coli* | 4 (8·16%) | 2 (4·88%) |
| *Candida* spp. | 3 (6·12%) | 2 (4·88%) |
| MRCNS | 2 (4·08%) | 1 (2·44%) |
| Others | 8 (16·33%) | 8 (19·51%) |

MRCNS: methicillin-resistant coagulase-negative staphylococci
