# Supplementary material for: Efficacy of aqueous olanexidine compared with alcohol-based chlorhexidine for surgical skin antisepsis regarding the incidence of surgical-site infections in clean-contaminated surgery: a randomized superiority trial
Source: Br J Surg. 2025 Apr 1;112(4):znaf065. doi: 10.1093/bjs/znaf065 (PMC12475902; doi:10.1093/bjs/znaf065)
Supplement: znaf065_Supplementary_Data [file znaf065_supplementary_data.zip › CONSORT_diagram.doc]

**BJS CONSORT diagram**

Assessed for eligibility

*n =830*

Randomized

*n* =700

Excluded *n =130*

Did not meet inclusion criteria *n =105*

Refused to participate *n =25*

**Olanexidine**

Allocated to intervention *n =350*

Received intervention *n =* 347

Did not receive intervention *n =3*

**Chlorhexidine-alcohol**

Allocated to intervention *n =350*

Received intervention *n =* 345

Did not receive intervention *n =5*

Lost to follow-up *n =4*

Clean instead of clean contaminated surgery *n* =1

*Died or reoperation during 30-day follow-up n=3*

Lost to follow-up *n =4*

Clean instead of clean contaminated surgery *n* =1

*Died or reoperation during 30-day follow-up n=3*

Analysed (FAS) *n =347*

Analysed (per-protocol) *n =343*

Analysed (FAS) *n =345*

Analysed (per-protocol) *n =341*

Analysis

Enrolment

Allocation

Follow-up
